# Characterisation of SapYZUs891@Fe/Mn-MOF Provides Insight into the Selection of Temperate Phage and Nanozyme for the Rapid and Sensitive Colourimetric Detection of Viable *Staphylococcus aureus* in Food Products

**DOI:** 10.3390/foods14213726

**Published:** 2025-10-30

**Authors:** Wenyuan Zhou, Wenjuan Li, Yeling Han, Aiping Deng, Yajie Li, Qin Hu, Lei Yuan, Guoqiang Zhu, Zhenquan Yang

**Affiliations:** 1 College of Food Science and Engineering, Yangzhou University, Yangzhou 225127, China; wenyuanzhou@yzu.edu.cn (W.Z.); 18256690807@163.com (W.L.); 15234912095@163.com (Y.H.); 18805192405@163.com (A.D.); m19505271215@163.com (Y.L.); qinhu@yzu.edu.cn (Q.H.); leiyuan@yzu.edu.cn (L.Y.); 2 College of Veterinary Medicine, Yangzhou University, Yangzhou 225009, China; 3 Jiangsu Key Laboratory of Zoonosis, Yangzhou University, Yangzhou 225009, China; 4 Key Laboratory of Catering Food Processing and Safety Control, Yangzhou University, Yangzhou 225127, China

**Keywords:** *Staphylococcus*, bacteriophage, oxidase mimic, colourimetric determination, food samples

## Abstract

Although phage@nanozymes have proven to be a rapid, precise, and cost-effective method for detecting pathogens in food, the basis of phage and nanozyme selection remains poorly understood. In this study, a novel colourimetric biosensor utilising the temperate phage SapYZUs891 and an Fe/Mn-MOF nanozyme was constructed and assessed for its efficacy in detecting *Staphylococcus aureus* in food products. Notably, SapYZUs891 exhibited a high titre, broad host range, and strong pH and thermal stability. Moreover, the bimetallic Fe/Mn-MOF nanozyme exhibited an enhanced oxidase-mimicking ability, greater affinity, and a higher reaction rate. The biosensor had a detection time of 19 min, a detection limit of 69 CFU/mL, and a recovery rate between 92.52% and 121.48%, signifying its high reliability and accuracy in identifying *S. aureus*. This sensor distinguishes between viable and non-viable bacteria and demonstrates resistance to interferent bacterial and food compounds, likely attributable to the particular receptor-binding proteins of SapYZUs891 that bind to the teichoic acid wall on the *S. aureus*. These results indicated that the SapYZUs891@Fe/Mn-MOF is suitable for the rapid visual assessment of *S. aureus*. Moreover, the highly sensitive and specific detection system holds significant potential for extended application in on-site screening of *S. aureus* contamination within food processing environments.

## 1. Introduction

*Staphylococcus aureus* is a common foodborne pathogen [1] and produces exotoxins responsible for severe outbreaks of staphylococcal food poisoning (SFP) [2]. The genetic determinants encoding these exotoxins exhibit extreme mobility and are readily disseminated among viable bacterial populations through horizontal gene transfer mechanisms involving diverse mobile genetic elements [3]. Owing to the prevalence of *S. aureus* in diverse food categories, *S. aureus* poisoning has emerged as a significant public health hazard in numerous nations [4,5]. Over the past 20 years, 12,139 SFP infections have been reported in the United States and 10,163 in China, according to data from the Centers for Disease Control and Prevention [6]. Furthermore, inadequate hygiene practices during the production, sale, and consumption of ready-to-eat (RTE) foods facilitate *S. aureus* contamination [7]. Consequently, rapid, efficient, and precise identification of viable *S. aureus* in food is crucial for food safety and human health.

Biosensors that utilise colourimetric nanozymes demonstrate significant advantages in biomolecular analysis owing to their operational simplicity, rapid detection capabilities, and exceptional molecular specificity. These systems offer economical solutions for pathogen monitoring and are promising diagnostic tools for food applications [8]. Furthermore, as components of colourimetric biosensors, nanozymes exhibit outstanding attributes, such as cost-effectiveness, ease of mass production, impressive resilience under adverse conditions, and strong enzyme-like activity [9,10,11]. Metal–organic frameworks (MOFs) are an important category of hybrid crystalline materials characterised by periodic network structures formed through the coordination-driven self-assembly of metal-containing nodes (including single metal ions or polynuclear clusters) with polytopic organic bridging ligands. Owing to their structural and functional properties, MOFs exhibit great potential as nanozyme candidates for catalytic applications [12]. As the most abundant redox-active elements in the Earth’s crust [13], iron (Fe) and manganese (Mn) meet the requirements for low-cost and readily available materials in foodborne pathogen detection methods. Furthermore, the disparate redox potentials of these two metals can synergistically enhance the oxidase-like properties when manganese and iron ions are incorporated into Fe-MOFs, which have great application potential in the colourimetric detection of *S. aureus* in different food samples [14]. However, rational screening strategies and the underlying mechanisms of nanozymes for colourimetric detection of pathogens in food systems remain to be elucidated.

Biorecognition elements are essential components of biosensors and must exhibit remarkable specificity, selectivity, and sensitivity to analyte molecules [15]. Bacteriophages (phages) are a class of viruses that specifically target bacteria without causing any harm to the human body and offer distinct advantages owing to their abundance in nature, robust proliferative capabilities, cost-effectiveness, and tolerance to pH and temperature variations [16]. Because of their unique biological properties, bacteriophages have emerged as promising recognition components for biosensing applications. Recent studies have successfully employed nanozyme platforms immobilised on lytic phages for foodborne pathogen monitoring, particularly for *S. aureus* and *Cronobacter sakazakii* [17,18]. However, because virulent phages may lyse host bacteria during the detection process, temperate phages are more suitable as recognition elements for pathogen detection. Therefore, screening temperate phage candidates with effective selection basis is essential for developing targeted phage-based detection methods for foodborne pathogens [19].

**Scheme 1:**
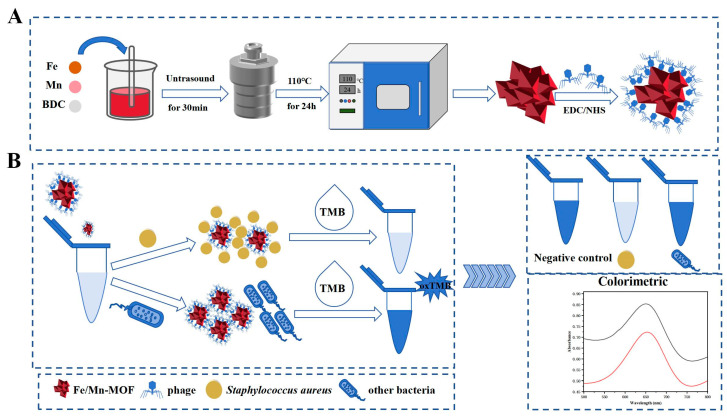
Synthesis of SapYZUs891@Fe/Mn-MOF (**A**) and colourimetric detection of *S. aureus* using the biosensor (**B**).

In this study, Fe/Mn bimetallic MOF and three other nanozymes (His-Fe_3_O_4_@Cu, Fe_1_Co_1_O, and CuO-POM) were prepared, and a utility assay indicated that Fe/Mn-MOF is the only nanozyme suitable as a transducer in the phage@nanozyme biosensor. Subsequently, a temperate *S. aureus* phage was isolated, and its biological features (one-step growth, host range, pH stability, temperature stability, and adsorption rate) and genetic characterisations revealed its suitability as bacteriophage-based biosensors. *S. aureus* temperate phage SapYZUs891 and Fe/Mn-MOF nanozyme were used to fabricate a colourimetric biosensor, SapYZUs891@Fe/Mn-MOF (Scheme 1). The characterisation and utility of SapYZUs891@Fe/Mn-MOF were evaluated to (i) develop a highly sensitive and specific colourimetric technique for the identification of *S. aureus* in RTE meals, and (ii) understand the chemical, biological, and genetic properties of a colourimetric biosensor suitable for pathogen detection in food.

## 2. Materials and Methods

### 2.1. Chemicals and Reagents

The chemical reagents and experimental materials used in this study are described in the Supplementary Materials.

### 2.2. Bacterial Strains and Culture Conditions

Sixty-three *S. aureus* strains [20] and the other 10 standard isolates used in this study are listed in Table S1.

### 2.3. Phage SapYZUs891 Isolation

SapYZUs891 was induced, isolated, and purified following previously published protocols, with some optimized modifications [21,22,23]. In brief, *S. aureus* strain YZUstau89 (2 × 10^7^ CFU/mL) was treated with mitomycin C (0.5 µg/mL, Shanghai Aladdin Biochemical Technology Co., Ltd., Shanghai, China) for 6 h. Sample collection began three hours after induction and continued at 60-min intervals. For each time point, 300 µL of the bacterial culture was carefully extracted and processed through sequential purification steps. The collected samples initially underwent centrifugation to separate cellular debris, followed by filtration of the resultant supernatant through sterile 0.22 μm membrane filters (Millex, Merck Millipore, Tullagreen, Carrigtwohill, Co. Cork, Ireland) to ensure complete removal of bacterial cells and large particulates. Phage titres were evaluated using the double-layer agar plate method.

### 2.4. Biological and Genomic Characterisation of the Isolated S. aureus Temperate Phage

Phages were purified as described previously [20]. The biological characterisation of SapYZUs891, including its morphology, one-step growth curve, and temperature and pH stability, was conducted in accordance with a previously established methodology [15,21].

Host specificity of the bacteriophage was evaluated through spot assays performed on 63 *S. aureus* isolates following established methodologies [3]. Briefly, bacterial cultures (100 μL) were mixed with 0.7% agar-supplemented Luria–Bertani (LB) medium and overlaid on 1.4% agar plates. After solidification, 10 μL aliquots of phage lysate were spotted onto the bacterial lawn. Following incubation at 37 °C for 6–8 h in an inverted position, phage susceptibility was determined by analysing spot clarity. Phage lytic activity was quantified based on plaque formation and zone transparency, with a grading system of strong lysis (+++), moderate lysis (++), weak lysis (+), or no lysis (−).

The complete genomic sequence of SapYZUs891 was determined through next-generation sequencing, followed by bioinformatics prediction and functional annotation using established protocols [24]. The whole genome sequence of SapYZ891 was submitted to GenBank (accession number PQ567070).

### 2.5. Protein Modeling and Molecular Docking of Phage Tail Proteins ORF3 and ORF65

The amino acid sequences of the phage tail proteins ORF3 and ORF65 of phage SapYZUs891 were retrieved and subjected to homology modeling using the SWISS-MODEL (https://swissmodel.expasy.org) (accessed on 6 September 2025) server as previously described [25]. The template for each domain was selected based on sequence identity and coverage (Table S2). The molecular docking between the phage tail proteins and N-acetylglucosamine (GlcNAc) was performed as previously described [26]. Briefly, the GlcNAc ligand was drawn and optimized using the MarvinSketch program and YASARA Structure. The protein–ligand docking was performed using Autodock4.2 (https://autodock.scripps.edu/download-autodock4/) (accessed on 6 September 2025) [25]. The docking results were analyzed using PyMOL version 3.1 (https://pymol.org/2/) (accessed on 6 September 2025) and Discovery Studio (Dassault Group, Paris, France).

### 2.6. Immobilisation of SapYZUs891 on the Nanozymes

Nanozymes (Fe/Mn-MOF, His-Fe_3_O_4_@Cu, Fe_1_Co_1_O, and CuO-POM) were prepared as previously described [27,28,29,30]. Specifically, 0.27 g of FeCl_3_·6H_2_O and 0.166 g of terephthalic acid were obtained from Shanghai Macklin Biochemical Co. Ltd. (Shanghai, China), and ultrasonically dissolved in 10 mL of N, N-dimethylformamide (DMF) to form a precursor solution of Fe/Mn-MOF. A 0.20g amount of MnCl_2_·4H_2_O (1.0 mM), serving as the manganese source, was introduced into this solution, followed by additional ultrasonication for homogenization. The mixture was then transferred to a 25 mL Teflon-lined autoclave and maintained at 110 °C for 24 h. After the reaction, the resulting product was collected, thoroughly washed with deionized water and ethanol, and dried under ambient conditions to obtain the final material. The immobilisation of phage SapYZUs891 on nanozymes was conducted using the carbodiimide cross-linking technique [31].

### 2.7. Utility of SapYZUs891@nanozymes in S. aureus Detection

The catalytic performance of SapYZUs891@nanozyme (SapYZUs891@Fe/Mn-MOF, SapYZUs891@His-Fe_3_O_4_@Cu, SapYZUs891@Fe_1_Co_1_O, and SapYZUs891@CuO-POM) was assessed by spectrophotometric analysis using TMB and H_2_O_2_ (Shanghai Aladdin Biochemical Technology Co., Ltd., Shanghai, China) as an enzymatic substrate [3,21]. Briefly, 100 μL aliquots of bacterial suspension (*S. aureus* YZUstau76, 10^1^–10^8^ CFU/mL) and each SapYZUs891@nanozyme solution were thoroughly mixed, respectively. This was followed by the addition of 2700 μL of acetate buffer and incubation at 25 °C. After introducing 100 μL of ethanolic TMB (5 mM) and H_2_O_2_ solution, the reaction mixture was immediately subjected to absorbance measurement.

### 2.8. Characterisation of SapYZUs891@Fe/Mn-MOF

The Supplementary Materials provides detailed characterisation data for SapYZUs891@Fe/Mn-MOF.

### 2.9. Oxidase-like Activity of SapYZUs891@Fe/Mn-MOF

The catalytic performance of SapYZUs891@Fe/Mn-MOF as an oxidase mimic was evaluated by spectrophotometric analysis of chromogenic substrates (TMB, ABTS, and OPD) at 652 nm using UV-vis spectroscopy (UV-3200, MAPADA, Shanghai, China) [32]. Specifically, a reaction system containing 100 μL of the nanocomposite and 100 μL of 5 mM TMB in 2800 μL of acetate buffer (0.2 M, pH 4.0) was monitored for 16 min at 25 °C. The ideal detection conditions were established by analysing the impact of varying pH levels, temperatures, and concentrations of TMB and SapYZUs891@Fe/Mn-MOF on the reaction system [33].

### 2.10. Selectivity, Anti-Interference, and Live/Dead Cell Discrimination

To systematically evaluate the specificity and interference resistance of the developed biosensing platform, 10 distinct bacterial species were selected for comprehensive analysis, following previously published experimental protocols [20]. Additionally, *S. aureus* was heated at 100 °C for 10 min to eradicate live cells. Living and thermally treated *S. aureus* cells were used to evaluate the capacity of the biosensor to distinguish between viable and dead bacteria [34].

### 2.11. Effects of Food Preservatives, NaCl, and pH on SapYZUs891@Fe/Mn-MOF

To evaluate the performance of the proposed biosensor system under various conditions, we examined the effects of multiple food factors by following established protocols [21]. The study incorporated (1) five common food additives at specified concentrations (potassium sorbate, 0.0075%; sodium pyrophosphate, 0.5%; sodium hexametaphosphate, 0.5%; sodium tripolyphosphate, 0.5%; and disodium succinate, 2%); (2) sodium chloride solutions ranging from 2% to 10% (in 2% increments); and (3) acetate buffers with pH values spanning 3–9. These components were added to LB broth containing *S. aureus* strain YZUstau76 at controlled inoculum levels (3 × 10^3^, 3 × 10^5^, or 3 × 10^7^ CFU/mL). For biosensor testing, 100 μL aliquots of each sample were mixed with 2900 μL of detection solution composed of 100 μL SapYZU891@Fe/Mn-MOF nanocomposite, 2700 μL acetate buffer (pH 4.0), and 100 μL TMB substrate.

### 2.12. Application of SapYZUs891@Fe/Mn-MOF for Testing RTE Food Samples

Five commercially available RTE food products were obtained from a local supermarket in Yangzhou, Jiangsu Province, China, and served as representative samples for this study. The selected items included braised beef with tomatoes, curry beef, braised pork, braised chicken with mushroom, and braised duck. The food samples were processed and sterilised according to previously established protocols [18,35]. Briefly, each food sample was subjected to thermal treatment at 110 °C for sterilisation, cooling to room temperature (25 °C), and refrigeration at 4 °C for preservation. For experimental analysis, prepared samples were artificially contaminated with *S. aureus* at varying concentrations (3 × 10^3^, 3 × 10^5^, or 3 × 10^7^ CFU/mL). Bacterial detection was performed using the newly developed biosensor platform and conventional colony-counting techniques.

### 2.13. The Detection Mechanisms of SapYZUs891@Fe/Mn-MOF

Free radical-scavenging experiments were conducted using a previously established protocol [36] and its protocol was provided in the Supplementary Materials.

### 2.14. Adsorption Rate

The adsorption rates of the separated phage particles were assessed using a previously established method [21] and its details were provided in the Supplementary Materials.

### 2.15. Statistical Analysis

Statistical analyses were conducted using three independent replicates for each experimental condition to ensure reliability. Quantitative results are presented as mean values with corresponding standard deviations (mean ± SD). Microsoft Excel 2021 and Origin 2021 software were used for the statistical analysis and graphical representation. Significant differences were determined using Duncan’s test and with a significance test at *p*-value of ≤0.05 [37].

## 3. Results and Discussion

### 3.1. Isolation and Characterisation of Temperate Phage SapYZUs891

**Figure 1:**
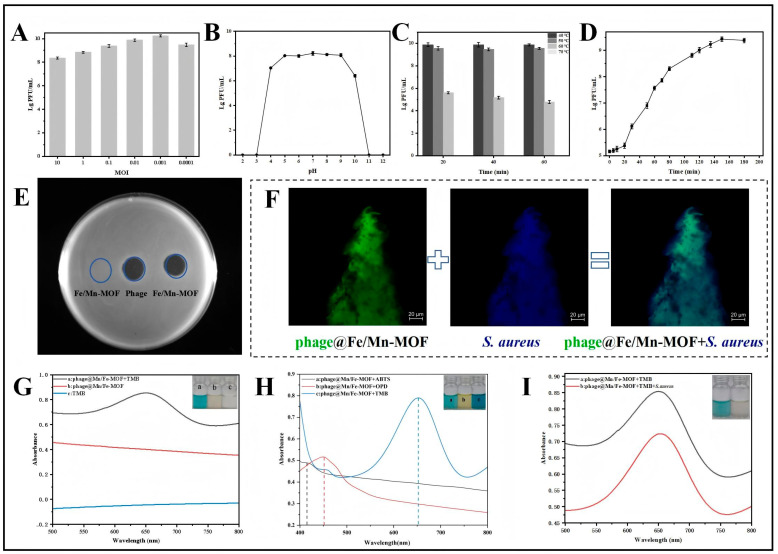
Phage titres (**A**): one-step growth curve (**B**); pH stability (**C**); and thermostability (**D**) of the phage SapYZUs891; spot tests of SapYZUs891, SapYZUs891@Fe/Mn-MOF, and Fe/Mn-MOF (**E**); laser-scanning confocal microscopy images of SapYZUs891@Fe/Mn-MOF alone and SapYZUs891@Fe/Mn-MOF + *S. aureus* (SapYZUs891@Fe/Mn-MOF was labelled with SYBR Green, and *S. aureus* was labelled with 4′,6-diamidino-2-phenylindole) (**F**); UV–vis spectra of the three reaction systems (**G**); UV–vis spectra of the four chromogenic systems (SapYZUs891@Fe/Mn-MOF + ABTS, SapYZUs891@Fe/Mn-MOF + OPD, SapYZUs891@Fe/Mn-MOF +TMB, and Fe/Mn-MOF + TMB) (**H**); and UV–vis spectra of the SapYZUs891@Fe/Mn-MOF + TMB chromogenic system, with or without *S. aureus* (the inset shows the corresponding real image) (**I**).

SapYZUs891 was successfully isolated from the *S. aureus* strain YZUstau89. Morphological examination demonstrated that the viral particles possessed an elliptical capsid structure connected to an elongated, rigid tail (Figure S1). Biological characterisation of SapYZUs891 indicated that the titre peaked at approximately 10^10^ plaque-forming units (PFU)/mL when the multiplicity of infection was 0.001 (Figure 1A). Comparative evaluation with previously characterised phages PHB21 (1 × 10^9^ PFU/mL) and SA13 (1 × 10^8^ PFU/mL) [38,39] revealed superior production yields for SapYZUs891, demonstrating that SapYZUs891 is produced more efficiently. SapYZUs891 exhibited relative stability following a 2-h incubation at 37 °C in buffer systems with pH levels ranging from 4 to 10 (Figure 1B), which surpasses the pH stability ranges of previously documented phages LSA2308 (pH 5–9), 1PHSA12 (pH 5–9), 2PHSA1 (pH 5–9), 4PHSA25 (pH 5–9), and JD419 (pH 6–8) [40,41,42], indicating that SapYZUs891 is appropriate for various intricate food processing settings. Similar to phage JD419 [42], SapYZUs891 exhibited stability when cultivated at 40–50 °C for durations of 20, 40, or 60 min (Figure 1C). The one-step growth curve exhibited a notable burst size of approximately 554 PFU/cell and an incubation duration of 5 min (Figure 1D). The bacteriophage SapYZUs891 demonstrated effective host cell lysis in 41 of the 63 tested *S. aureus* strains, corresponding to a host range specificity of 65.1% (Table S1). This broader spectrum of SapYZUs891 was compared to previously characterised temperate phages SapYZUs13 (22/47, 46.8%), SapYZUs14 (6/47, 12.8%), and SapYZUs16 (16/47, 36.0%) [21] (*p* ≤ 0.001), suggesting its enhanced host recognition [21,39]. The observed biological properties of SapYZUs891, particularly its broad host range and stability, support its potential utility as a highly specific biological recognition element targeting *S. aureus* on diagnostic platforms.

To comprehensively evaluate the potential of SapYZUs891 as a biorecognition molecule, its genome was analysed. SapYZUs891 is a double-stranded DNA phage with a genome size of 47,331 base pairs (Figure S2 and Table S3). The genome contains 68 open reading frames (ORFs) encoding holin, lysin, integrase, DNA polymerase, DNA-binding protein, DNA helicase, TerS, TerL, ClpP, and tail fibre proteins. Genomic integration between bacteriophages and their bacterial hosts is mediated by two key enzymatic components: a phage-encoded integrase and a ClpP protease system [43]. Identification of the integrase and ClpP genes in the SapYZUs891 genome confirmed its classification as a temperate phage capable of lysogenic replication. Host recognition by phages depends on their tail fibre proteins, which interact with the modified wall teichoic acids of *S. aureus*. The wall teichoic acids of *S. aureus* are decorated with either β-1,4-N-acetylglucosamine (catalysed by TarS) or α-1,4-N-acetylglucosamine (mediated by TarM) modifications, which are the targets of phage tail fibre proteins [44].

The predicted candidate receptor molecule (N-acetylglucosamine, GlcNAc) of *S. aureus* was molecularly docked with the optimized structure of phage SapYZUs891 tail proteins (Figure S3). The AutoDock Vina program was used, with the grid box covering the entire potential binding region, and multiple docking runs were performed to obtain a large number of binding conformations. Through cluster analysis of all docking results, multiple high-affinity binding sites were successfully identified, and key amino acid residues interacting with the receptor molecule were clarified (Table S2). These residues reveal the structural basis for protein-receptor binding. It is worth noting that the proteins encoded by the tail protein genes ORF 3 and ORF 65 exhibit 100% amino acid identity to phage tail proteins (WP_428704856.1 and HBI9298856.1, respectively). Studies have demonstrated that members of this protein family specifically recognize and bind to wall teichoic acids on the *S. aureus* cell wall as their receptor [45,46]. This finding is highly consistent with the host range of our phage, indicating that the tail proteins play a potent role in the recognition of phage towards *S. aureus*. Further studies will involve structural and functional analysis, through protein expression and interaction assays, to define the critical determinants and mechanism of WTA-specific recognition by the bacteriophage SapYZUs891 RBP. Moreover, this study suggests that the high SapYZUs891 titre may result from its unique genomic repertoire, particularly genes involved in nucleic acid metabolism. Genome annotation revealed the presence of multiple replication-associated genes encoding DNA polymerases, helix-destabilising proteins, and helicase enzymes in the SapYZUs891 genome, which collectively enhance virion production efficiency [47]. These molecular characteristics are associated with host-recognition specificity and efficient progeny phage replication, further suggesting that SapYZUs891 is an ideal biological recognition component for phage-based detection.

### 3.2. Characterisation of Materials

**Figure 2:**
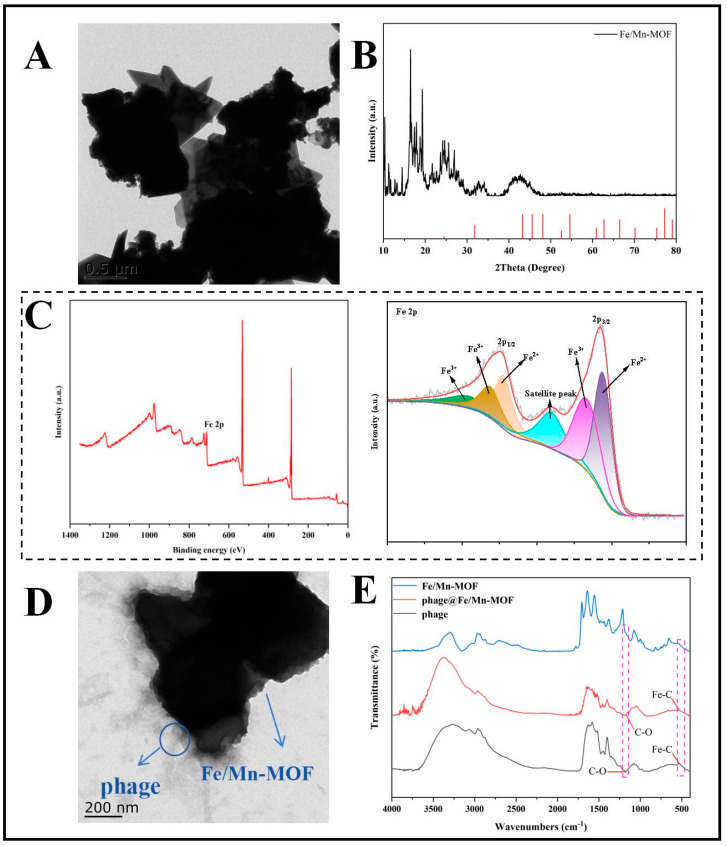
TEM image of Fe/Mn-MOF (**A**), X-ray diffraction pattern (**B**), and X-ray photoelectron spectroscopy spectrum (**C**) of Fe/Mn-MOF; transmission electron microscopy image of SapYZUs891@Fe/Mn-MOF (**D**); and Fourier-transform infrared spectroscopy curves of the phage SapYZUs891, Fe/Mn-MOF nanozyme, and SapYZUs891@Fe/Mn-MOF (**E**).

TEM images indicated that the synthesised Fe/Mn-MOF particles were approximately 500 nm in size and had a relatively decentralised distribution (Figure 2A). Crystalline characterisation of the synthesised Fe/Mn-MOF was performed using X-ray diffraction (XRD) analysis (Figure 2B). The diffraction profile displays sharp, well-defined peaks with high intensities, demonstrating excellent crystallinity. A comparison with the standard card confirmed the close correspondence between the experimental pattern and the standard diffraction profile (JCPDS No. 03-1180). Elemental composition analysis via X-ray photoelectron spectroscopy (XPS) detected characteristic signals corresponding to Fe, Mn, and carbon in a survey scan of the Fe/Mn-MOF sample. The full spectral data confirm the presence of these key constituent elements in the synthesised material (Figure 2C). The 2p_3/2_ and 2p_1/2_ peaks of Fe were resolved into two distinct sets: peaks at 729.73 eV, 726.44 eV, and 713.69 eV were attributed to Fe^3+^, while those at 724.57 eV and 711.25 eV were assigned to Fe^2+^. Two mixed valence states (Fe^2+^ and Fe^3+^) facilitate redox reactions. The XPS results indicated that the extent of Mn diffusion to the surface was minimal (<0.1% of the surface composition) [48]. The energy-dispersive X-ray spectroscopy (EDX) spectrum of the Fe/Mn-MOF demonstrated a homogeneous distribution of Fe and Mn (Figure S4). A previous study suggested that bimetallic catalysts provide superior catalytic efficacy and stability compared with single metals because of their distinctive interfacial impact, synergistic effect, and electron transport [49]. Moreover, metal ion doping can strengthen the active sites and improve catalytic performance [50]. These results imply that the Fe/Mn-MOF nanoparticles were successfully synthesised and have potential applications in *S. aureus* detection.

### 3.3. The Successful Immobilisation of SapYZUs891@Fe/Mn-MOF via Covalent Bonding

Following the immobilisation of SapYZUs891 onto the Fe/Mn-MOF, the TEM images indicated that the phage heads of SapYZUs891 were situated near the Fe/Mn-MOF surface, with tails protruding outwards (Figure 2D). The successful immobilisation of phages on the nanozyme was validated using Fourier-transform infrared (FTIR) spectroscopy (Figure 2E). FTIR analysis of both SapYZUs891 and SapYZUs891@Fe/Mn-MOF revealed distinct absorption bands at 536 cm^−1^ and 1178 cm^−1^, corresponding to C-O and Fe-C vibrational modes, respectively. The observed infrared absorption bands presumably originated from molecular interactions between the capsid proteins of SapYZUs891 and the Fe/Mn-MOF nanostructure. Variations in peak intensity reflect distinct functional group characteristics, and the identified bond vibration patterns provide additional evidence of interfacial interactions between the bacteriophage and nanozyme components [34]. These spectroscopic findings collectively verified the formation of SapYZUs891@Fe/Mn-MOF via covalent bonding.

### 3.4. Biological Activity and Oxidase-Mimicking Ability of SapYZUs891@Fe/Mn-MOF

The biological activity of SapYZUs891@Fe/Mn-MOF was systematically evaluated in this study. These results confirmed the preservation of the antimicrobial activity of SapYZUs891@Fe/Mn-MOF against *S. aureus* (Figure 1E). Fluorescence-based characterisation employing dual-labelling techniques (SYBR Green-stained SapYZUs891@Fe/Mn-MOF and DAPI-labelled *S. aureus* strain YZUstau76) provided further evidence of the biological activity of SapYZUs891@Fe/Mn-MOF (Figure 1F). The images of the SapYZUs891@Fe/Mn-MOF composite sample and strain YZUstau76 exhibited a consistent distribution of speckled green and blue fluorescent signals. These results collectively demonstrate that the phage SapYZUs891 maintains its biological specificity after immobilisation on the MOF matrix.

The enzymatic activity of SapYZUs891@Fe/Mn-MOF was assessed using TMB as the chromogenic reagent [15,51]. In the absence of H_2_O_2_, SapYZUs891@Fe/Mn-MOF oxidised TMB, producing blue light with a high A_652_ value; however, no colour alteration was observed in the control experiment (Figure 1G). These findings indicate that SapYZUs891@Fe/Mn-MOF shows oxidase-like activity. Furthermore, SapYZUs891@Fe/Mn-MOF promoted the oxidation of colourless ABTS and OPD compounds, yielding green (416 nm) and yellow (448 nm) derivatives, respectively (Figure 1H). Notably, TMB demonstrated superior chromogenic properties as an oxidising substrate and was selected as the standard indicator for all subsequent colourimetric assays. The enzymatic activity of bare Fe/Mn-MOF was also investigated. Importantly, the conjugation of bacteriophage components to the nanozyme surface preserved catalytic functionality, with no statistically significant reduction in enzymatic performance observed following phage immobilisation. This maintenance of catalytic efficiency suggests that the biological modification process does not interfere with the active sites responsible for the enzyme-mimicking properties of the nanomaterials.

Kinetic analysis of SapYZUs891@Fe/Mn-MOF nanozyme was performed using TMB as the substrate. Kinetic analysis through Michaelis-Menten and Lineweaver-Burk transformations yielded maximum reaction velocity (*V_max_*) and Michaelis constant (*K_m_*) values of 2.35 × 10^−8^ M/s and 0.45 mM, respectively (Figure S5A and S5B). The Michaelis constant (*K_m_*) serves as a quantitative measure of enzyme–substrate binding affinity, where smaller values correspond to stronger interactions between the catalytic site and the substrate [51]. Comparative kinetic analysis demonstrated that SapYZUs891@Fe/Mn-MOF exhibited a significantly reduced *K_m_* value for TMB oxidation compared to other phage@nanozyme systems, including Fe-MOF@SalmpYZU47 [52] and Cu-MOF@PpZDSS02 [53] (Table S4). Regarding catalytic efficiency, the maximal reaction velocity (*V_max_*) reflects the theoretical turnover rate when the enzyme’s active sites are fully saturated with the substrate [51]. Notably, SapYZUs891@Fe/Mn-MOF achieved superior *V_max_* performance in TMB conversion compared to reference nanomaterials such as MOF-808 and Fe-N-C single-atom catalysts (Table S4). These kinetic parameters collectively highlight the enhanced substrate affinity and catalytic capacity of SapYZUs891@Fe/Mn-MOF.

The colourimetric sensing capability of SapYZUs891@Fe/Mn-MOF for *S. aureus* was evaluated by spectrophotometric analysis at 652 nm. Comparative measurements were performed on the reaction system both before and after the introduction of the *S. aureus* strain YZUstau76. The initial spectral analysis revealed a characteristic absorption band in the absence of bacterial cells (Figure 1I). Notably, a marked decrease in absorbance at 652 nm was observed after the addition of the bacterial solution. These experimental results demonstrate the effective application of SapYZUs891@Fe/Mn-MOF as a chromogenic detection platform for *S. aureus*, which is consistent with the findings of a recent study [18].

### 3.5. Optimisation of the Testing Conditions

The impact of varying the pH level, SapYZUs891@Fe/Mn-MOF concentration, TMB, and temperature on the catalytic activity was examined [15]. The catalytic activity of SapYZUs891@Fe/Mn-MOF reached its maximum at pH 4.0 (Figure S6). The catalytic activity increased linearly with an increase in SapYZUs891@Fe/Mn-MOF concentration (Figure S7). Considering catalytic efficiency, reaction kinetics, cost-effectiveness, and catalyst dosage comprehensively, a volume of 100 μL of the SapYZUs891@Fe/Mn-MOF nanocomposite was selected, aligning with established protocols reported in prior research [18]. Spectrophotometric analysis revealed a linear correlation between the absorbance of the detection system and the concentration of the chromogenic substrate, TMB (Figure S8). To minimise reagent consumption and maintain detection sensitivity, a TMB concentration of 5 mM was identified as optimal for subsequent assays. Furthermore, the temperature-dependent catalytic activity of the biosensor was investigated within a range of 20 to 40 °C (Figure S9). The results revealed a progressive enhancement in the absorbance with increasing temperature. However, to ensure experimental reproducibility and minimise potential thermal degradation effects, a standardised reaction temperature of 25 °C was adopted for all subsequent analyses.

The absorbance of the SapYZUs891@Fe/Mn-MOF chromogenic system was similar (~0.43) in 24 min; thus, the optimal recognition time was set to 3 min (Figure S10). Kinetic analysis of the colourimetric reaction revealed distinct temporal patterns in the SapYZUs891@Fe/Mn-MOF system with or without *S. aureus* (Figure S11A). The control system (without *S. aureus*) demonstrated a progressive enhancement in optical density, whereas samples containing the target pathogen exhibited gradual attenuation of the absorbance signals. The quantitative evaluation of the absorbance ratio demonstrated a consistent decline during the initial 16-min reaction period, after which the values reached equilibrium (Figure S11B). Based on these kinetic profiles, an optimal reaction time of 16 min was established for subsequent analytical procedures. Therefore, the developed biosensor exhibited superior performance, with a total assay time of only 19 min, compared to traditional culture methods (24–120 h) [54], PCR assays (240 min) [55], as well as enzyme-linked immunosorbent assay (90 min) [56]. Furthermore, it demonstrates significant superiority over other phage@nanozyme methodologies (Table S5), including AuPt@ vB_YepM_ZN18 (40 min) [57] and AuNPs@T156 (80 min) [58]. The biosensing system demonstrated significant advantages in speed and operational simplicity, making it suitable for rapid screening in food processing environments and testing laboratories. Consequently, SapYZUs891@Fe/Mn-MOF serves as a more efficient colourimetric biosensor for the detection of *S. aureus*.

Through systematic optimisation studies, the experimental parameters yielding the maximum detection efficiency of the SapYZUs891@Fe/Mn-MOF-based biosensing platform were comprehensively determined. The established protocol incorporates the following carefully optimised conditions: a reaction temperature maintained at 25 °C, an acidic buffer system adjusted to pH 4.0, a final concentration of 100 μL of SapYZUs891@Fe/Mn-MOF, 5 mM TMB, a 3 min incubation time, and a 16 min reaction time.

### 3.6. Sensitivity, Specificity, Anti-Interference, and Ability to Differentiate Live/Dead Bacteria of SapYZUs891@Fe/Mn-MOF

**Figure 3:**
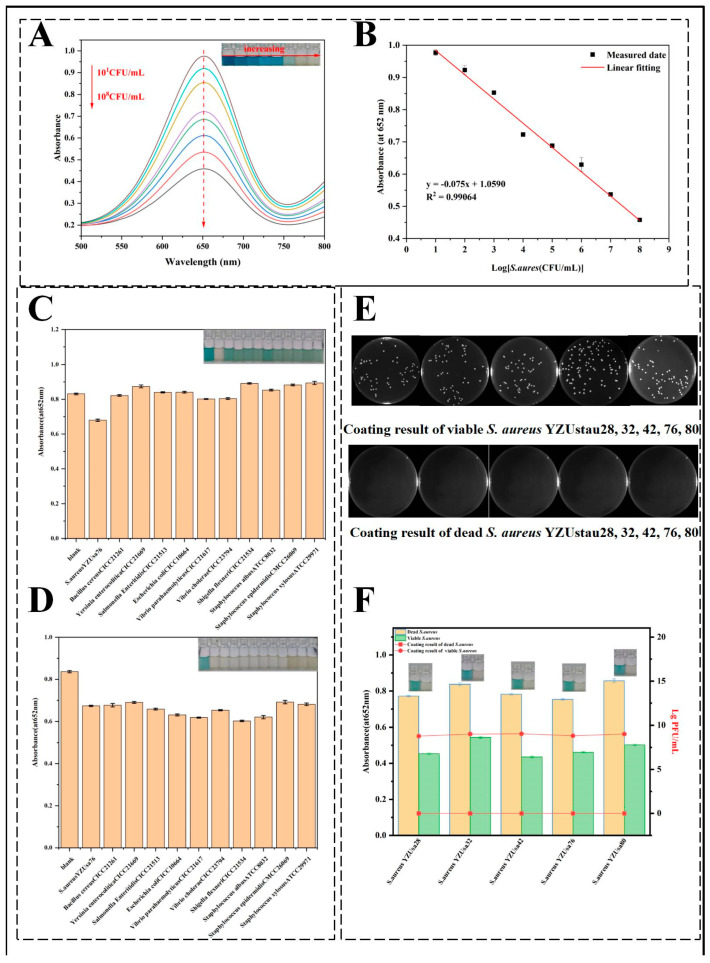
UV–vis spectra of the Fe/Mn-MOF + TMB chromogenic system with different concentrations of *S. aureus* (**A**); linear fitting between the absorbance of the chromogenic system at 652 nm and the logarithm of the *S. aureus* concentration (**B**); selectivity (**C**) and anti-interference capacity (**D**) of the Fe/Mn-MOF system; coating of dead and viable *S. aureus* cells (**E**); and performance of Fe/Mn-MOF in determining viable *S. aureus* counts (**F**).

As shown in Figure 3A,B, the linear range and detection limit of SapYZUs891@Fe/Mn-MOF were 30–3.0 × 10^8^ CFU/mL and 69 CFU/mL, respectively. Table S5 shows that the limit of detection of our proposed biosensor was superior to those of other detection methods, including PCR (1.0 × 10^2^ CFU/mL) [56], lateral flow assay (1.0 × 10^3^ CFU/mL) [59], long-period fiber grating (224 CFU/mL) [60], vancocin/IgG (290 CFU/mL) [61], nanobodies (1.4 × 10^5^ CFU/mL) [62], and antibody/AuNP/MNPs (1.5 × 10^3^ CFU/mL) [63]. Therefore, our SapYZUs891@Fe/Mn-MOF biosensor exhibited a broader linear range and a reduced detection limit. The analytical performance of biosensors for *S. aureus* detection depends critically on two key parameters: selectivity and resistance to interference. To rigorously evaluate these characteristics, 10 bacterial species commonly found in co-contaminated food matrices were selected for comparative analysis. As shown in Figure 3C, the absorbance value of the SapYZUs891@Fe/Mn-MOF system exhibited minimal variation upon the introduction of interfering bacteria, demonstrating the superior selectivity of the biosensor for *S. aureus*. Concerning the anti-interference capability (Figure 3D), none of the 10 competing bacterial strains produced detectable signal interference during *S. aureus* quantification, confirming the analytical reliability of this system in complex microbial environments. The selectivity of this phage-based system likely stems from specific molecular interactions between the phage and its bacterial host [21].

In addition, the SapYZUs891@Fe/Mn-MOF biosensor successfully identified live *S. aureus* cells but ignored dead cells (Figure 3E,F). This result is likely due to the degradation or structural alteration of wall teichoic acids on non-viable *S. aureus* cells, thereby preventing special chemical bonding between the phage and *S. aureus*. Consequently, the biosensor constructed in this study targeted live *S. aureus* cells and was not affected by other bacterial species and dead *S. aureus*.

### 3.7. The Detection Performance of SapYZUs891@Fe/Mn-MOF in Real Food Samples

**Figure 4:**
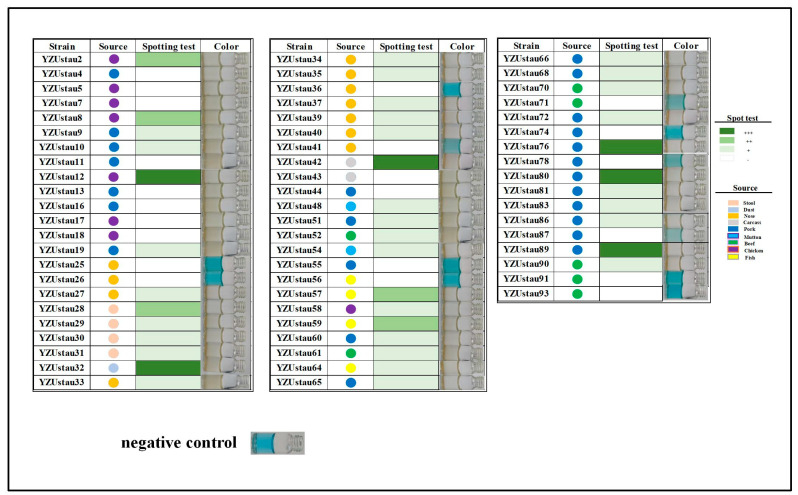
Host range of SapYZUs891 and the ability of SapYZUs891@Fe/Mn-MOF to detect 63 *S. aureus* isolates. The results of spot test were classified into four groups: +++, clear and translucent plaque; ++, slightly turbid plaque; +, plaque turbidity;–, no plaque.

To investigate the utility of SapYZUs891@Fe/Mn-MOF, 63 strains of *S. aureus* from various sources (stool, dust, nose, carcase, pork, mutton, beef, chicken, and fish) were used. The proposed biosensor demonstrated excellent detection performance for all strains (63/63, 100%) (Figure 4). These 63 strains comprised 13 sequence types (STs), including ST9 (15/63, 23.8%), ST398 (13/63, 20.6%), and ST7 (9/63, 14.3%) (Table S1). These results demonstrate that the SapYZUs891@Fe/Mn-MOF biosensor is capable of detecting viable *S. aureus* contamination in diverse food products, although it cannot distinguish between *S. aureus* isolates of different genotypes. Multilocus sequence typing (MLST) is widely used to investigate the epidemiology of *S. aureus*, and the distribution of MLST types varies by region and source, providing an essential tool for epidemiological tracking of contamination sources [64,65]. Furthermore, strain-specific variations in *S. aureus* often involve modifications of cell wall components, such as glycosylation or alanylation of wall teichoic acids, which may alter the binding efficiency of phage-derived receptor proteins and lead to fluctuations in detection sensitivity [66]. In addition, biofilm formation by certain high-virulence strains can mask surface binding sites, potentially resulting in false-negative signals or reduced detection efficacy [67]. To address these limitations and enhance genotype-specific identification, we will integrate genetic markers (CRISPR-Cas systems) or molecular probes into the biosensing platform in our future study [68].

Furthermore, SapYZUs891@Fe/Mn-MOF showed superior efficacy in identifying *S. aureus* strains that SapYZUs891 failed to lyse. The SapYZUs891 phage’s recognition of *S. aureus* hosts was validated by evaluating its adsorption rate to non-host *S. aureus*, which ranged from 2.75% to 52.34% (Figure S12). Thus, adsorption between phages and target bacteria plays a potent role in the recognition of phage-based chromogenic systems, which should be considered the basis for phage selection. This study was limited to the number of identified *S. aureus* isolates, and our future studies will focus on developing a biosensor utilising a phage cocktail to reduce the possibility of false negatives.

To further assess the *S. aureus* detection efficacy of SapYZUs891@Fe/Mn-MOF, the influence of prevalent food preservatives, sodium chloride, and pH was examined. As shown in Figure S13A,C, the SapYZUs891@Fe/Mn-MOF biosensing platform demonstrated robust performance in quantifying *S. aureus* concentrations, exhibiting remarkable resilience against potential interfering factors, including various food preservatives, sodium chloride concentrations, and pH variations. These results are consistent with our previous research findings [21], which probably resulted from the dilution of the test samples by almost 30-fold, significantly diminishing the impact of interfering chemicals. In addition, the enzyme activity of SapYZUs891@Fe/Mn-MOF was stable for 30 days, which is a satisfactory duration of stability (Figure S14). The detection proficiency of SapYZUs891@Fe/Mn-MOF was also tested on real Chinese RTE food samples, and the calculated mean recovery percentage was in the range of 92.52–121.48% (Figure S13 and Table S6), indicating the high quantitative accuracy and reliable detection capability of our colourimetric platform for *S. aureus* in complex food matrices. This robust recovery profile supports the real-world applicability of SapYZUs891@Fe/Mn-MOF.

### 3.8. The Detection Mechanism of SapYZUs891@Fe/Mn-MOF

**Figure 5:**
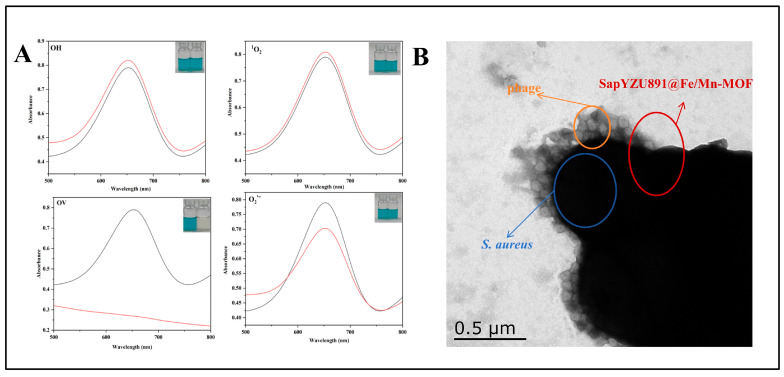
The influence of the concentration of IPA, L-Trp, EDTA•2Na, and BQ on the free radical trapping experiments (**A**), and TEM images of SapYZUs891@Fe/Mn-MOF + *S. aureus* (**B**).

To elucidate the catalytic mechanism of the biosensor, IPA, *_L_*-Trp, EDTA•2Na, and BQ were employed as quenchers for •OH, ^1^O_2_, oxygen vacancies, and O_2_^•–^ free radicals, respectively (Figure 5A). The colour diminished progressively, and the UV–vis absorption spectrum signal significantly decreased following the addition of EDTA•2Na, suggesting that oxygen vacancies were pivotal in TMB oxidation within this system. Electron paramagnetic resonance (EPR) characterization revealed a significant decrease in oxygen vacancy signals upon introduction of *S. aureus* hosts to SapYZUs891@Fe/Mn-MOF, indicating that the bacterial cells attenuated the signal intensity and consequently reduced the formation of oxTMB (Figure S15). Furthermore, TEM images demonstrated that SapYZUs891@Fe/Mn-MOF was encircled by *S. aureus* cells, which preserved their biological integrity and were not lysed by SapYZUs891 (Figure 5B). Consequently, we assumed that the diminished oxidase-like activity of SapYZUs891@Fe/Mn-MOF upon the introduction of *S. aureus* host was attributable to the obstruction of oxygen vacancies by the *S. aureus* host [21]. These findings align with previously reported mechanisms where the addition of thiram (THR) was shown to impede effective contact between Cu(II) active sites and catalytic substrates, leading to suppressed laccase-mimicking activity [69].

The recognition, detection, and tracking of foodborne pathogens play a crucial role in the food industry, and colourimetric methods are attractive for the direct visual assessment of pathogens. In this study, Fe/Mn-MOF nanozyme and phage SapYZUs891 were selected to construct a chromogenic detection system. Compared to other reported chromogenic detection methods (Ag/Mn_3_O_4_ [70], GNR-SH-NB [71], and P-CLISA [72]), SapYZUs891@Fe/Mn-MOF is characterised as a more rapid, cost-effective, selective, and easily prepared food monitoring system for viable *S. aureus*. A previous study suggested that a biosensor based on virulent phages might lyse the target pathogen during detection, leading to unstable detection signals and false-negative results [56]. Therefore, the *S. aureus* temperate phage SapYZUs891, with a relatively broad host range, high pH, thermal stability, and attractive genetic properties, was selected. Moreover, the nanomaterial used as a transducer is another key element affecting the capability of the biosensor [73]. The Fe/Mn-MOF nanozyme was easily constructed using common materials and exhibited attractive enzyme–substrate binding affinity and catalytic capacity. Notably, the effect of SapYZUs891@Fe/Mn-MOF on food samples was evaluated using 63 *S. aureus* isolates from distinct sources and other interfering pathogens, thus confirming its utility in food applications. Consequently, we propose that these data will provide insights into the construction of a phage-based chromogenic detection system. Future studies should develop convenient nanodevices based on phage cocktails and enhance their utility by using samples from real food production processes.

## 4. Conclusions

In conclusion, a colourimetric biosensor, SapYZUs891@Fe/Mn-MOF, was prepared using the phage SapYZUs891, which exhibited a high titre, broad host range, and strong pH and thermal stability. The bimetallic catalyst Fe/Mn-MOF, exhibiting enhanced oxidase-mimicking ability, greater affinity, and reaction rate. SapYZUs891@Fe/Mn-MOF enables rapid detection of live *S. aureus* in food matrices, exhibiting high sensitivity and selectivity. The oxidase activity of SapYZUs891@Fe/Mn-MOF markedly decreased upon interaction with *S. aureus*, which was likely attributable to the obstruction of oxygen vacancies on the surface of the nanozyme. Furthermore, SapYZUs891@Fe/Mn-MOF demonstrated the ability to identify *S. aureus* hosts from diverse origins and *S. aureus* isolates that SapYZUs891 was unable to lyse, possibly due to the unique receptor-binding proteins on the tail fibre of SapYZUs891. These findings suggest that the SapYZUs891@Fe/Mn-MOF colourimetric system is a promising biosensor for the detection of live *S. aureus* isolates in food matrices. The SapYZUs891@Fe/Mn-MOF colourimetric system provides an innovative direction for utilizing phages as cost-effective diagnostic reagents in the food industry, enabling rapid, reliable, visual, and practical detection of bacterial contamination. Future efforts should focus on leveraging this phage-based colorimetric system to develop commercial detection platforms (e.g., microfluidic chips, portable devices), thereby enabling practical, on-site screening for the food industry.

## Data Availability

The original contributions presented in the study are included in the article/Supplementary Materials, further inquiries can be directed to the corresponding author.

## Supplementary Materials

The following supporting information can be downloaded at: https://www.mdpi.com/article/10.3390/foods14213726/s1, Figure S1: TEM image of the phage SapYZUs891; Figure S2: Gene structure of phage SapYZUs891; Figure S3: Interaction between SapYZUs891 tail Proteins ORF3 (A) and ORF65 (B) with the N-acetylglucosamine molecule; Figure S4: Energy-dispersive X-ray spectroscopy mapping of Fe/Mn-MOF; Figure S5: Steady-state kinetic analysis of SapYZUs891@Fe/Mn-MOF simulated oxidase activity: (A) The Michaelis-Menten curve. (B) The Lineweaver-Burk plot.; Figure S6: Effect of buffer solution pH on the SapYZUs891@Fe/Mn-MOF + TMB chromogenic system; Figure S7: Effect of SapYZUs891@Fe/Mn-MOF amount on the SapYZUs891@Fe/Mn-MOF + TMB chromogenic system; Figure S8: Effect of buffer solution TMB concentration on the SapYZUs891@Fe/Mn-MOF + TMB chromogenic system; Figure S9: Effect of temperature on the SapYZUs891@Fe/Mn-MOF + TMB chromogenic system; Figure S10: Effect of incubation time between SapYZUs891@Fe/Mn-MOF and *S. aureus* on the SapYZUs891@Fe/Mn-MOF + TMB chromogenic system; Figure S11: Effect of reaction time on the chromogenic systems with/without *S. aureus* (A) and reaction time-dependent ratio of absorbances at 652 nm of chromogenic systems with/without *S. aureus* (B); Figure S12: Adsorption rate of phage SapYZUs891 to *S. aureus* that cannot lysis; Figure S13: Effects of common food additives (A), different concentrations of NaCl (B), and pH values (C) on the colorimetric detection of *S. aureus*; and the performance of SapYZU891@Fe/Mn-MOF for the detection of *S. aureus* in real food (D); Figure S14: 30 d of stability tests of SapYZUs891@Fe/Mn-MOF + TMB chromogenic system; Figure S15: Electron paramagnetic resonance spectrum of the captured free radicals by oxygen vacancy; Table S1: Host range of SapYZUs891; Table S2: Interacting residues and binding sites of SapYZUs891 tail proteins ORF3 and ORF65 with GlcNAc; Table S3: Genomic function annotation of SapYZUs891; Table S4: Comparison of the steady-state kinetic parameters for the oxidase-like activity of SapYZUs891@Fe/Mn-MOF and other nanozymes; Table S5: Comparison between SapYZUs891@Fe/Mn-MOF chromogenic system and previously reported studies; Table S6: The effect of different food additives on the SapYZUs891@Fe/Mn-MOF chromogenic system in culture.
