# Supplementary material for: Characterisation of SapYZUs891@Fe/Mn-MOF Provides Insight into the Selection of Temperate Phage and Nanozyme for the Rapid and Sensitive Colourimetric Detection of Viable Staphylococcus aureus in Food Products
Source: Foods. 2025 Oct 30;14(21):3726. doi: 10.3390/foods14213726 (PMC12610485; doi:10.3390/foods14213726)
Supplement: Supplementary file 1 [file foods-14-03726-s001.zip › foods-3893094-supplementary.pdf]

Supplementary Material

Characterisation of SapYZUs891@Fe/Mn-MOF Provides Insight into the Selection of Temperate Phage and Nanozyme for the Rapid and Sensitive Colourimetric Detection of Viable *Staphylococcus aureus* in Food Products

Wenyuan Zhou [1,2,3,4], Wenjuan Li [1], Yeling Han [1], Aiping Deng [1], Yajie Li [1], Qin Hu [1,4], Lei Yuan [1,4], Guoqiang Zhu [2,*] and Zhenquan Yang [1,3,4,*]

[1] College of Food Science and Engineering, Yangzhou University, Yangzhou 225127, China; wenyuanzhou@yzu.edu.cn (W.Z.); 18256690807@163.com (W.L.); 15234912095@163.com (Y.H.); 18805192405@163.com (A.D.); m19505271215@163.com (Y.L.); qinhu@yzu.edu.cn (Q.H.); leiyuan@yzu.edu.cn (L.Y.)
[2] College of Veterinary Medicine, Yangzhou University, Yangzhou 225009, China
[3] Jiangsu Key Laboratory of Zoonosis, Yangzhou University, Yangzhou 225009, China
[4] Key Laboratory of Catering Food Processing and Safety Control, Yangzhou University, Yangzhou 225127, China
* Correspondence: yzgqzhu@yzu.edu.cn (G.Z.); yangzq5730@163.com (Z.Y.)

1. Materials and methods

1.1. Chemicals and reagents

The chemical reagents utilized in this study were obtained from two commercial suppliers. Manganese chloride ($MnCl_2 \cdot 4H_2O$), Ferric chloride hexahydrate ($FeCl_3 \cdot 6H_2O$), Terephthalic acid (BDC), dimethylformamide (DMF) were obtained from Shanghai Macklin Biochemical Co.,Ltd. Sodium acetate, glacial acetic acid (HAc), isopropyl alcohol (IPA), 3,3′,5,5′-tetramethylbenzidine (TMB), Nhydroxysuccinimide (NHS), 1-ethyl-3-(3-dimethylaminopropyl)-carbodiimide hydrochloride (EDC), and 2-(N-Morpholino)-ethanesulfonic acid monohydrate (MES), ethylenediaminetetraacetic acid disodium salt (EDTA•2Na), *p*-benzoquinone (BQ), and L-tryptophan (L-Trp) were obtained from Shanghai Aladdin Biochemical Technology Co., Ltd. (Shanghai, China).

1.2. Characterisation of SapYZUs891@Fe/Mn-MOF

The microstructure of Fe/Mn-MOF and SapYZUs891 was characterized by transmission electron microscopy (TEM, Tecnai 12, Netherlands) [1]. Crystal structure measurements were carried out on

an X-ray diffractometer (XRD, D8 Advance, Bruker AXS, Germany) [2]. The surface chemical elements and valence states of the samples were detected by X-ray photoelectron spectroscopy (XPS, Thermo Scientific Escalab 250Xi, USA) [3]. The fluorescence image of phage and bacteria was obtained by laser scanning confocal microscope (LSCM, LSM 880NLO, Carl Zeiss, Germany). Elemental mapping analysis was conducted using energy dispersive spectroscopy (EDS) [4]. Fourier transform infrared (FT-IR) spectra were recorded using FT-IR spectrometer (FT-IR, VERTEX 70 German Bruker). The UV–vis absorption spectra and absorbance were recorded on a UV–vis spectrophotometer (UV-3200, MAPADA, China). An electron paramagnetic resonance spectrometer (EPR, A300-10/12, Bruker, Germany) was used for determining the free radicals.

1.3. Determination of catalytic kinetic parameters

A gradient of substrate concentration of TMB (2.5, 5, 7.5, 10 and 12.5 mM) was set, and the reaction was detected within 5 min under optimal conditions, and the initial reaction rate was recorded. The kinetic parameters (K_m and V_{max}) were calculated based on the Michaelis-Menten equation [5].

$$\frac{1}{V} = \frac{K_m}{V_{max}[s]} + \frac{1}{V_{max}}$$

The V_{max} is the maximum reaction rate, K_m is the Michaelis constant, [s] is the substrate concentration, and V is the initial reaction rate.

1.4. Adsorption rate

The adsorption rates of the isolated bacteriophages were determined as previously described [6]. The lysate of isolated phages (5 mL) was mixed with an equal volume of the suspension of their *S. aureus* hosts (Multiplicity of infection (MOI) = 0.1). The mixture was then incubated at 37 °C for 15 min and centrifuged at 8000 rpm/min for 30 s and the supernatant was diluted and spotted onto a double-layer agar plate containing the lawns of host strains to determine the phage titer. Adsorption rate (%) = (initial phage titer −phage titer after incubation) / initial phage titer.

1.5. Study of the catalytic mechanisms of the oxidase-like activity of SapYZUs891@Fe/Mn-MOF

Free radical-scavenging experiments were conducted as previously described [7]. Isopropyl alcohol (IPA), *p*-benzoquinone (BQ), ethylenediaminetetraacetic acid disodium salt (EDTA•2Na) and L-tryptophan (L-Trp) were used as scavengers to eliminate hydroxyl radicals (•OH), superoxide anion radicals ($O_2^{\bullet -}$), oxygen vacancies, and singlet oxygen ($^1O_2$) from the reaction system. The absorption spectra were measured after the addition of the scavengers, and electron paramagnetic resonance was used to detect free radicals in the reaction system.

1.6. Stability of SapYZUs891@Fe/Mn-MOF under different pH values and temperatures

The stability of YZU891@Fe/Mn-MOF stored under different pH and temperature conditions was evaluated according to the protocols described in previous studies with some modifications [8,9]. For the pH stability evaluation, the YZU891@Fe/Mn-MOF were incubated in buffer solutions of different pH values (3–12) for 1 h, respectively. For the thermostability evaluation, the particles were incubated at 4–70 °C for 1 h, respectively. The catalytic activity was investigated using 100 μL of TMB (5 mM).

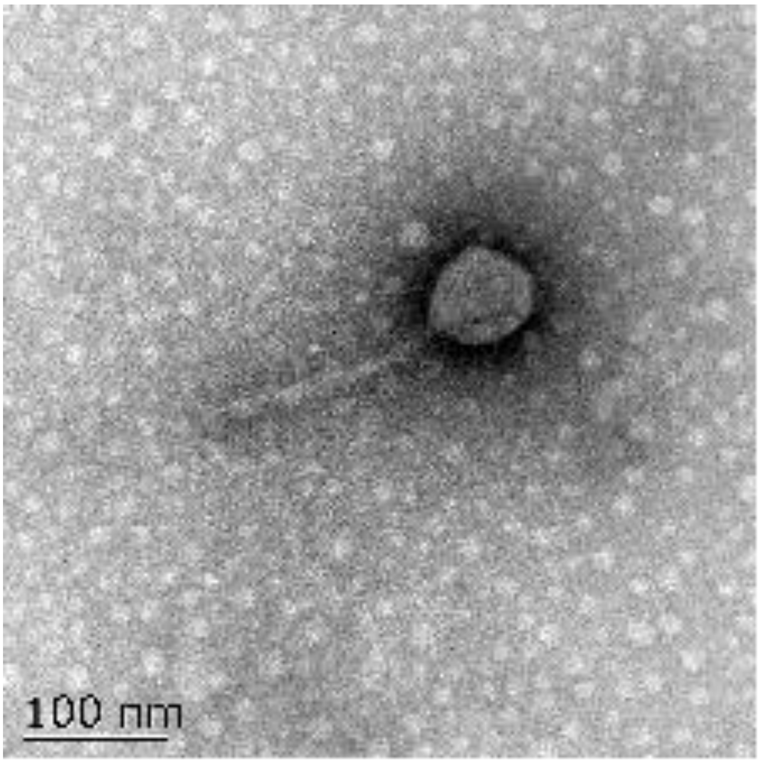

Figure S1. TEM image of the phage SapYZUs891.

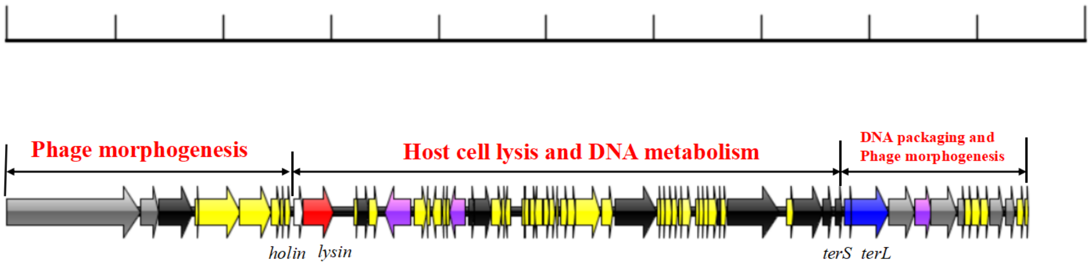

Figure S2. Gene structure of phage SapYZUs891.

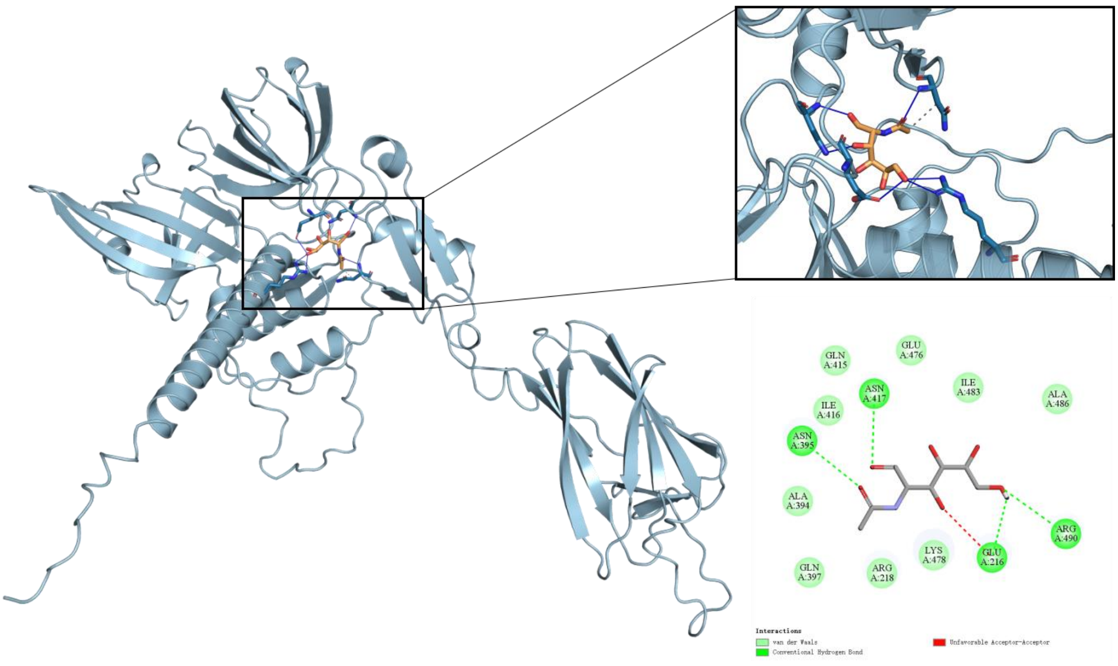

A

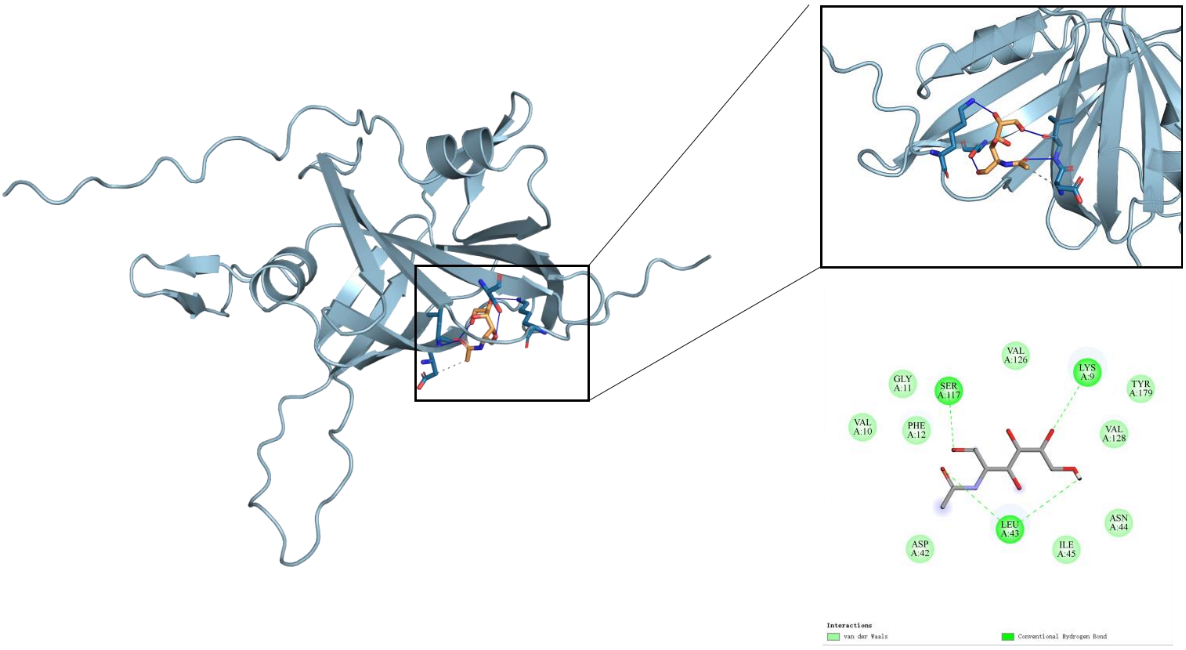

B

Figure S3. Interaction between SapYZUs891 tail Proteins ORF3 (A) and ORF65 (B) with the N-acetylglucosamine molecule.

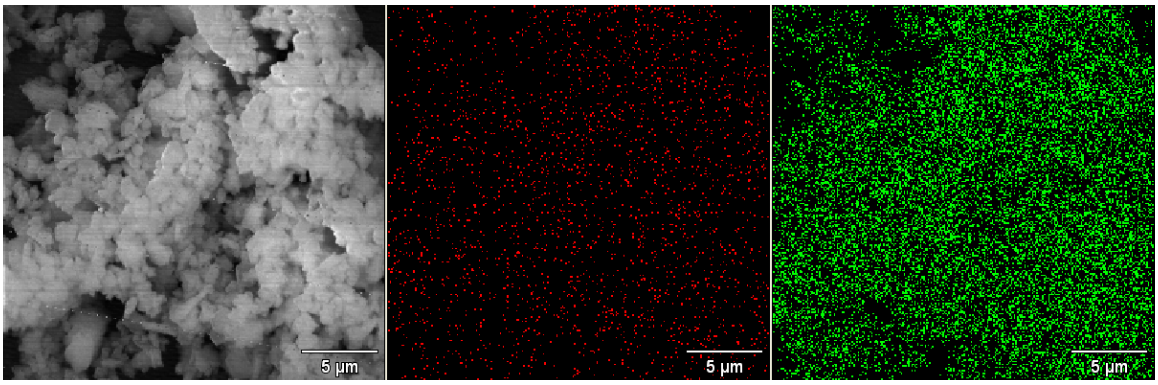

Figure S4. Energy-dispersive X-ray spectroscopy mapping of Fe/Mn-MOF.

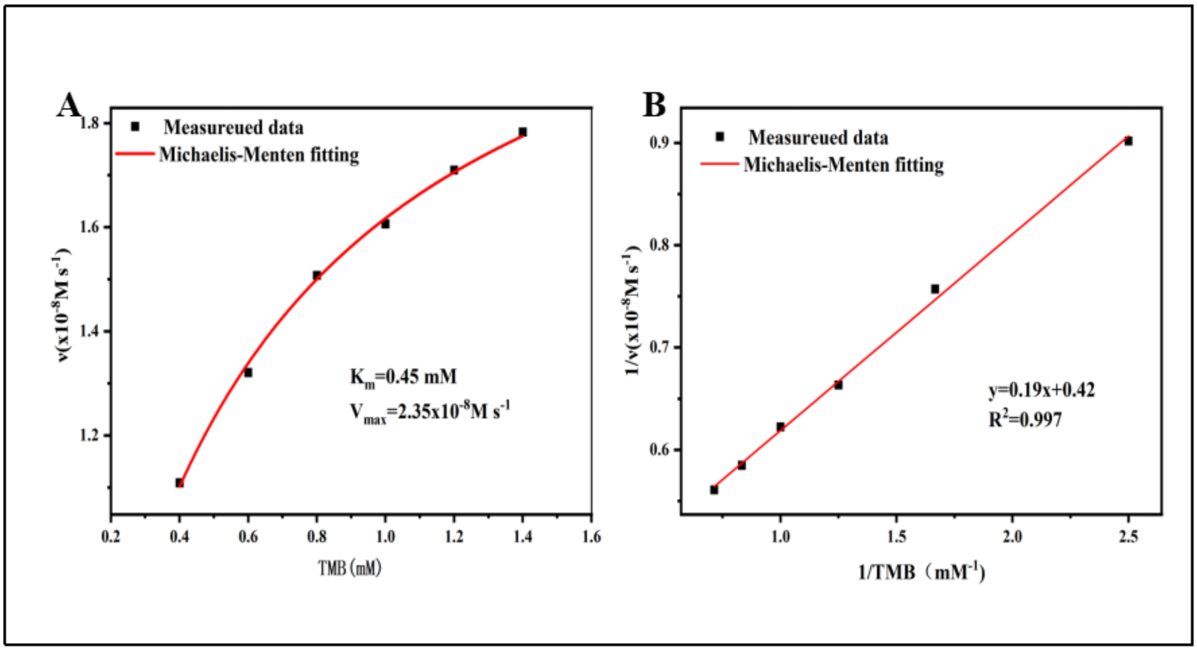

Figure S5. Steady-state kinetic analysis of SapYZUs891@Fe/Mn-MOF simulated oxidase activity: (A) The Michaelis-Menten curve. (B) The Lineweaver-Burk plot.

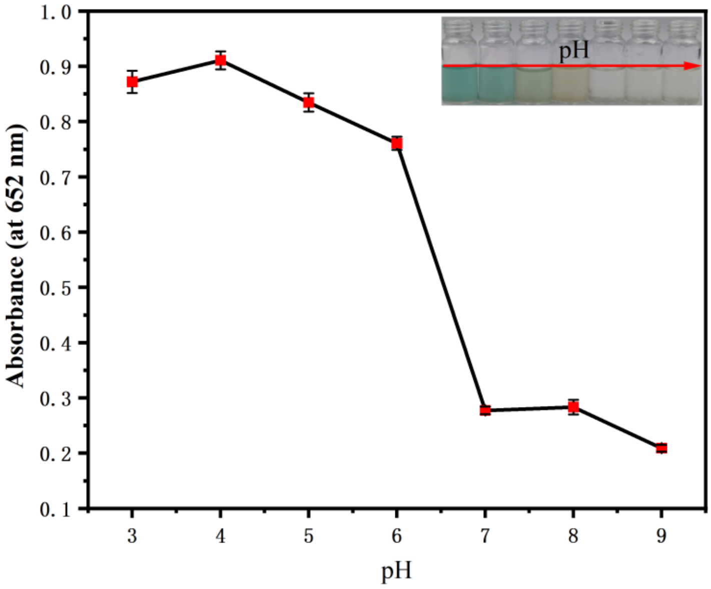

Figure S6. Effect of buffer solution pH on the SapYZUs891@Fe/Mn-MOF + TMB chromogenic system.

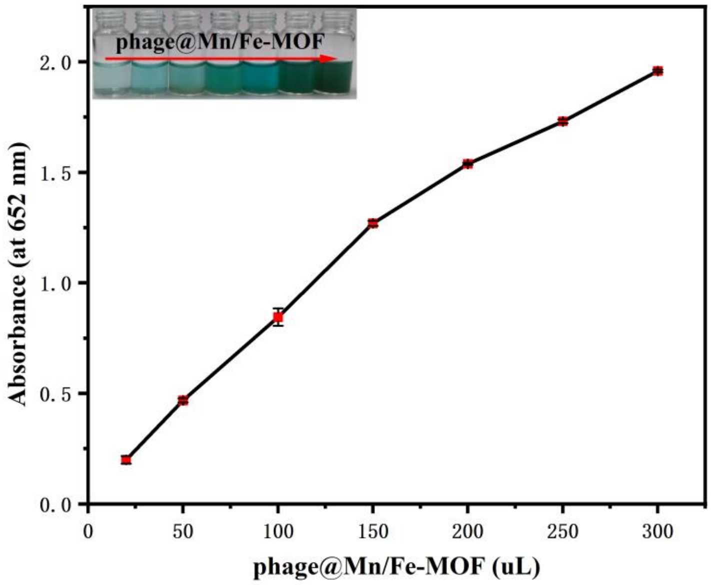

Figure S7. Effect of SapYZUs891@Fe/Mn-MOF amount on the SapYZUs891@Fe/Mn-MOF + TMB chromogenic system.

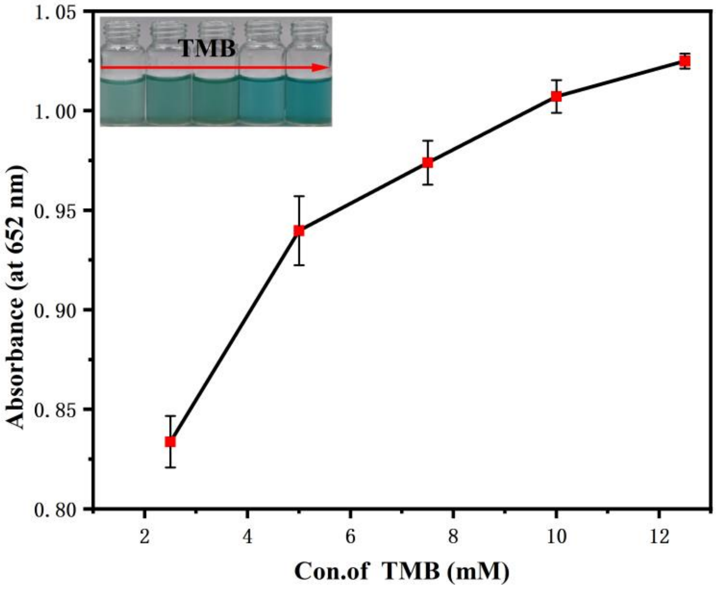

Figure S8. Effect of buffer solution TMB concentration on the SapYZUs891@Fe/Mn-MOF + TMB chromogenic system.

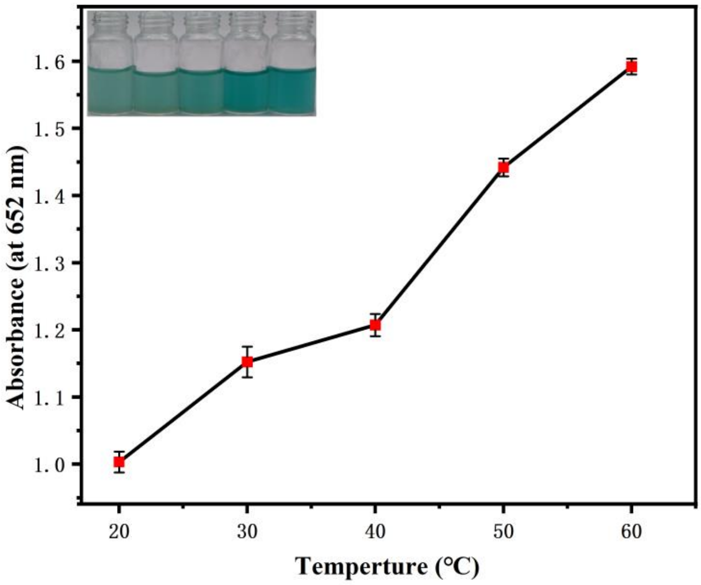

Figure S9. Effect of temperature on the SapYZUs891@Fe/Mn-MOF + TMB chromogenic system.

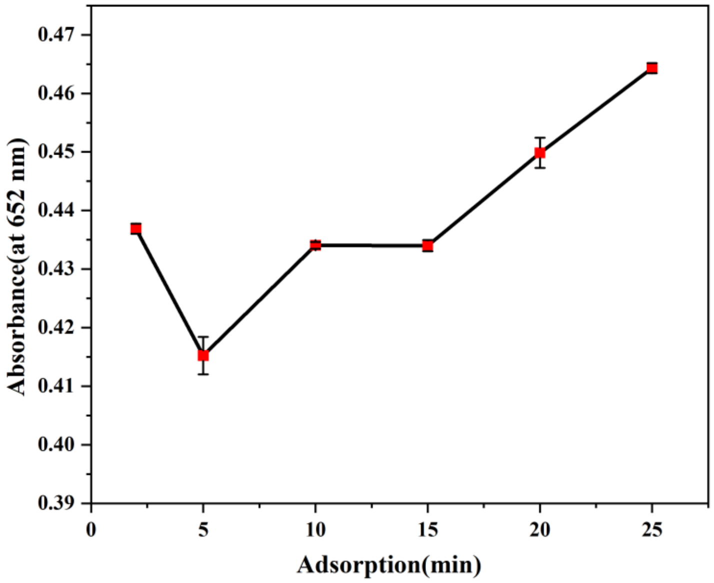

Figure S10. Effect of incubation time between SapYZUs891@Fe/Mn-MOF and *S. aureus* on the SapYZUs891@Fe/Mn-MOF + TMB chromogenic system.

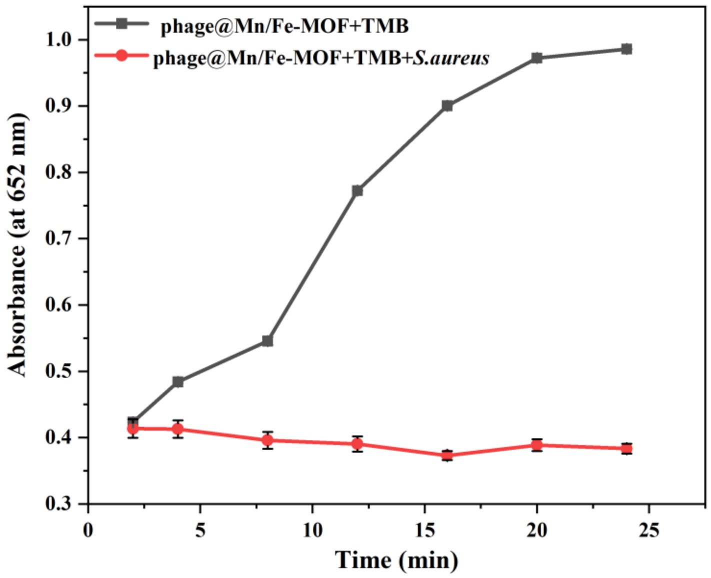

A

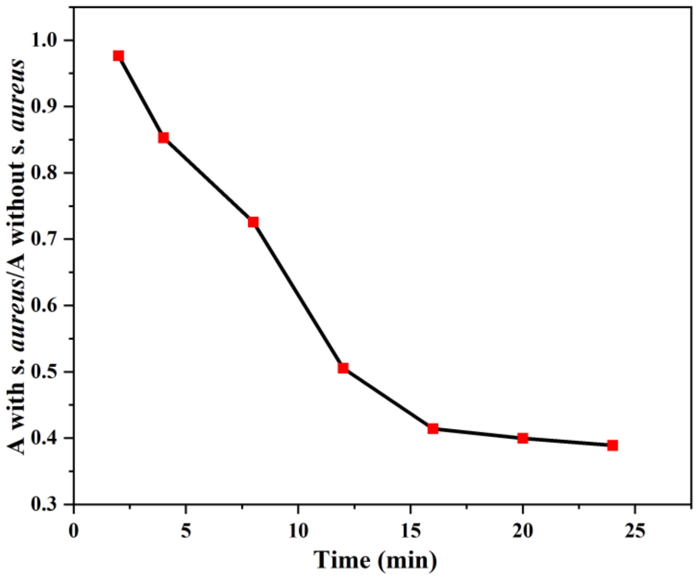

B

Figure S11. Effect of reaction time on the chromogenic systems with/without *S. aureus* (A) and reaction time-dependent ratio of absorbances at 652 nm of chromogenic systems with/without *S. aureus* (B).

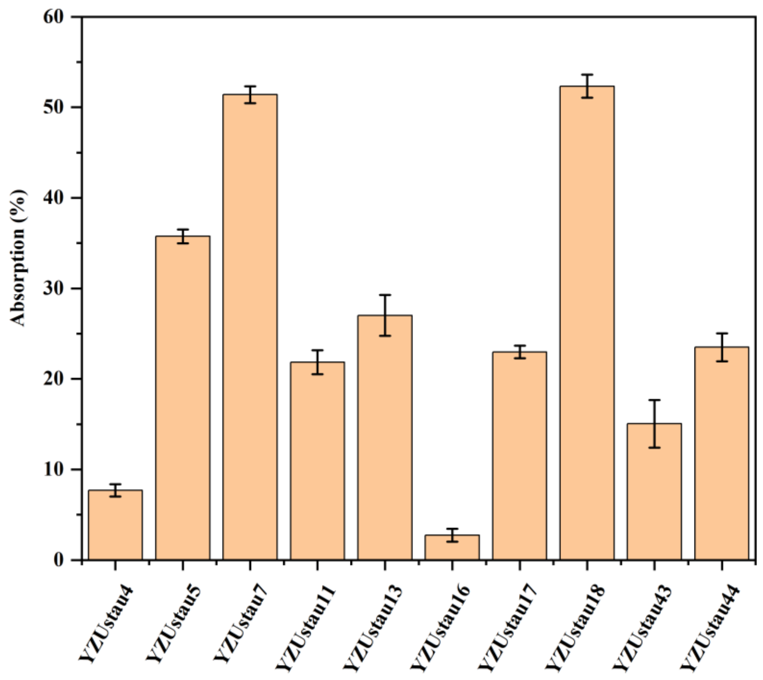

Figure S12. Adsorption rate of phage SapYZUs891 to *S.aureus* that cannot lysis.

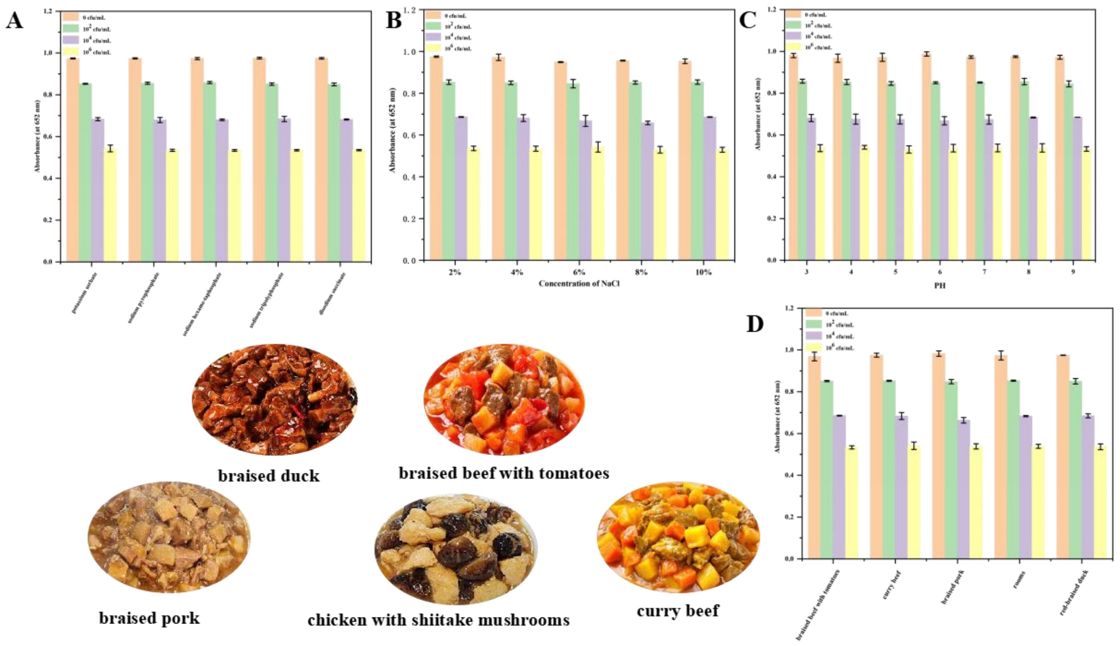

Figure S13. Effects of common food additives (A), different concentrations of NaCl (B), and pH values (C) on the colorimetric detection of *S. aureus*; the real food products used in this study (D); and the performance of SapYZU891@Fe/Mn-MOF for the detection of *S. aureus* in real food.

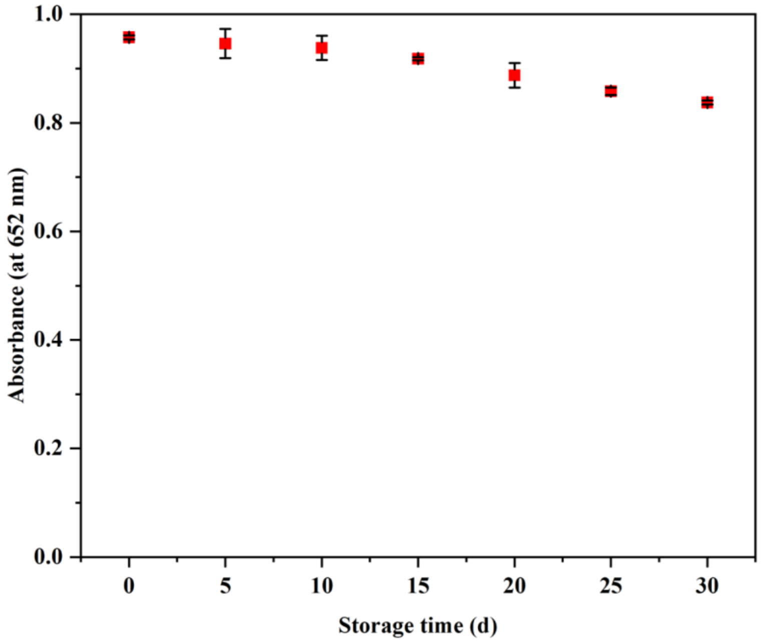

Figure S14. 30 d of stability tests of SapYZUs891@Fe/Mn-MOF + TMB chromogenic system.

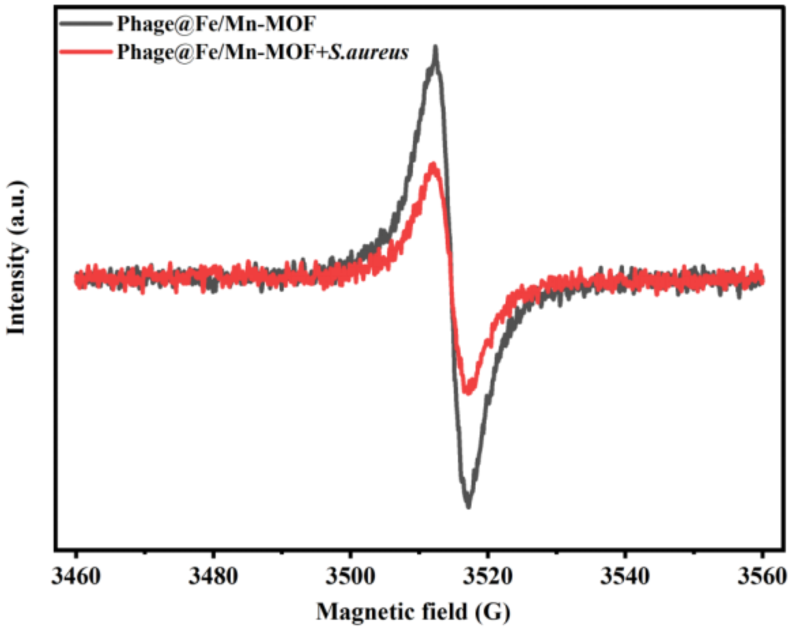

Figure S15. Electron paramagnetic resonance spectrum of the captured free radicals by oxygen vacancy.

Table S1. Host range of SapYZUs891.

No.	Strain	Species	Host	MASR or MSSR	MLST	Spotting test
1	YZUstau2	*S. aureus*	Chicken	MSSA	ST2990	++
2	YZUstau4	*S. aureus*	Pork	MSSA	ST7	-
3	YZUstau5	*S. aureus*	Chicken	MSSA	ST398	-
4	YZUstau7	*S. aureus*	Chicken	MSSA	ST7	-
5	YZUstau8	*S. aureus*	Chicken	MSSA	ST398	++
6	YZUstau9	*S. aureus*	Pork	MSSA	ST398	+
7	YZUstau10	*S. aureus*	Pork	MRSA	ST398	+
8	YZUstau11	*S. aureus*	Pork	MSSA	ST398	-
9	YZUstau12	*S. aureus*	Chicken	MSSA	UT	+++
10	YZUstau13	*S. aureus*	Pork	MSSA	ST398	-
11	YZUstau16	*S. aureus*	Pork	MSSA	ST188	-
12	YZUstau17	*S. aureus*	Chicken	MRSA	ST398	-
13	YZUstau18	*S. aureus*	Chicken	MRSA	ST398	-
14	YZUstau19	*S. aureus*	Pork	MSSA	ST8	+
15	YZUstau25	*S. aureus*	Pork	MSSA	ST9	-
16	YZUstau26	*S. aureus*	Pork	MSSA	ST7	-
17	YZUstau27	*S. aureus*	Pork	MSSA	ST9	+
18	YZUstau28	*S. aureus*	Pork	MSSA	ST9	++
19	YZUstau29	*S. aureus*	Pork	MSSA	ST9	+
20	YZUstau30	*S. aureus*	Pork	MSSA	ST9	+
21	YZUstau31	*S. aureus*	Pork	MSSA	ST9	+

22	YZUstau32	*S. aureus*	Pork	MSSA	ST8	+++
23	YZUstau33	*S. aureus*	Pork	MSSA	ST9	+
24	YZUstau34	*S. aureus*	Pork	MSSA	ST9	+
25	YZUstau35	*S. aureus*	Pork	MSSA	ST9	+
26	YZUstau36	*S. aureus*	Pork	MSSA	ST9	-
27	YZUstau37	*S. aureus*	Pork	MSSA	ST9	+
28	YZUstau39	*S. aureus*	Pork	MSSA	ST9	+
29	YZUstau40	*S. aureus*	Pork	MSSA	ST9	+
30	YZUstau41	*S. aureus*	Pork	MSSA	ST7	-
31	YZUstau42	*S. aureus*	Pork	MSSA	ST188	+++
32	YZUstau43	*S. aureus*	Pork	MSSA	ST188	-
33	YZUstau44	*S. aureus*	Pork	MSSA	ST188	-
34	YZUstau48	*S. aureus*	Mutton	MSSA	ST188	+
35	YZUstau51	*S. aureus*	Pork	MSSA	ST7	+
36	YZUstau52	*S. aureus*	Beef	MSSA	ST188	+
37	YZUstau54	*S. aureus*	Mutton	MSSA	ST7	+
38	YZUstau55	*S. aureus*	Pork	MSSA	ST398	-
39	YZUstau56	*S. aureus*	Fish	MSSA	ST1920	-
40	YZUstau57	*S. aureus*	Fish	MSSA	ST1	++
41	YZUstau58	*S. aureus*	Chicken	MSSA	ST7	+
42	YZUstau59	*S. aureus*	Fish	MSSA	ST1	++
43	YZUstau60	*S. aureus*	Pork	MSSA	ST8237	+
44	YZUstau61	*S. aureus*	Beef	MSSA	ST1	+

45	YZUstau64	*S. aureus*	Fish	MSSA	ST1	+
46	YZUstau65	*S. aureus*	Pork	MSSA	ST1920	+
47	YZUstau66	*S. aureus*	Pork	MSSA	ST1920	+
48	YZUstau68	*S. aureus*	Pork	MSSA	ST1920	+
49	YZUstau70	*S. aureus*	Beef	MSSA	ST7	+
50	YZUstau71	*S. aureus*	Beef	MSSA	ST1281	-
51	YZUstau72	*S. aureus*	Pork	MSSA	ST398	+
52	YZUstau74	*S. aureus*	Pork	MSSA	ST2990	-
53	YZUstau76	*S. aureus*	Pork	MSSA	ST1821	+++
54	YZUstau78	*S. aureus*	Pork	MSSA	ST630	-
55	YZUstau80	*S. aureus*	Pork	MSSA	ST398	+++
56	YZUstau81	*S. aureus*	Pork	MSSA	ST9	+
57	YZUstau83	*S. aureus*	Pork	MSSA	ST2990	+
58	YZUstau86	*S. aureus*	Pork	MSSA	ST398	+
59	YZUstau87	*S. aureus*	Pork	MSSA	ST9	-
60	YZUstau89	*S. aureus*	Pork	MSSA	ST1920	+++
61	YZUstau90	*S. aureus*	Beef	MSSA	ST398	+
62	YZUstau91	*S. aureus*	Beef	MSSA	ST7	-
63	YZUstau93	*S. aureus*	Beef	MSSA	UT	-
64	CICC 21669	*Yersinia enterocolitica*	-	-	-	-
65	CICC 21261	*Bacillus cereus*	-	-	-	-
66	CICC 21513	*Salmonella*	-	-	-	-

		Enteritidis				
67	CICC 21534	*Shigella flexneri*	-	-	-	-
68	CICC 21617	*Vibrio parahaemolyticus*	-	-	-	-
69	CICC 23794	*Vibrio cholerae*	-	-	-	-
70	CICC 10664	*Escherichia coli*	-	-	-	-
71	CMCC 26069	*Staphylococcus epidermidis*	-	-	-	-
72	ATCC 29971	*Staphylococcus xylosus*	-	-	-	-
73	ATCC 8032	*Staphylococcus albus*	-	-	-	-

"+++" indicates that the plaque is clear and transparent.

"++" indicates that the plaque is semitransparent.

"+" indicates that the plaque is shallow, and "-" indicates no plaque.

ATCC, American Type Culture Collection.

CICC, China Center of Industrial Culture Collection.

CMCC, National Center for Medical Culture Collections.

NT, non-typeable.

[a] No predicted ancestor contained ST7, ST59, ST398, ST1281, and ST2631, which was assigned to singleton.

Table S2. Interacting residues and binding sites of SapYZUs891 tail proteins ORF3 and ORF65 with GlcNAc.

Protein	Ligand	Template	Sequence identity	Coverage	% Residue in most favored regions of Ramachandran plot (MolProbity)	Interacting Amino Acids	Binding Affinity (kcal/mol)
ORF3	N-acetylglucosamine	Q2FYD0.1.A	99.43%	100%	96.19	ASN 395, ILE 416, GLN 415, ASN 417, GLU 476, ILE 483, ALA 486, ARG 490, GLU 216,LYS 478, ARG 218, GLN 397, ALA 394	-5.5
ORF65	N-acetylglucosamine	Q2G2K1.1.A	100%	100%	92.89	VAL 10， GLY 11, PHE 12, SER 117, VAL 126, LYS 9, TYR 179, VAL 128, ASN 44, ILE 45, LEU 43, ASP 42	-4.6

Table S3. Genomic function annotation of SapYZUs891.

ORFs	Start	End	Length(bp)	Strand	Protein(aa)	Putative function	Best phage homolog	Identity (%)	Accession no.
ORF1	1	6201	6201	+	2067	phage tail tape measure protein	Staphylococcus aureus	99.81	WP_257244512.1
ORF2	6201	7025	825	+	275	phage tail family protein	Staphylococcus aureus	100	HDE6118771.1
ORF3	7034	8617	1584	+	528	phage tail protein	Staphylococcus aureus	99.81	HDM8628118.1
ORF4	8712	8882	171	+	57	hypothetical protein	Staphylococcus aureus	100	WP_113590430.1
ORF5	8898	10808	1911	+	637	minor structural protein	Staphylococcus aureus	99.84	WP_031867387.1
ORF6	10775	12274	1500	+	500	BppU family baseplate upper protein	Staphylococcus phage SMSAP5	99.6	YP_007005720.1
ORF7	12274	12663	390	+	130	DUF2977 domain-containing protein	Staphylococcus aureus	100	WP_001166606.1
ORF8	12656	12820	165	+	55	XkdX family protein	Staphylococcus aureus	100	WP_000916022.1
ORF9	12866	13165	300	+	100	DUF2951 domain-containing protein	Staphylococcus aureus	98.99	HDG5980925.1
ORF10	13301	13738	438	+	146	holin	Staphylococcus aureus	100	WP_000354117.1
ORF11	13719	15164	1446	+	482	lysin	Staphylococcus aureus	100	WP_001148129.1
ORF12	16250	16795	546	+	182	hypothetical protein	Staphylococcus aureus	100	WP_224756974.1
ORF13	16785	17183	399	+	133	hypothetical protein	Staphylococcus aureus	99.24	WP_021285852.1
ORF14	17523	18728	1206	+	402	integrase	Staphylococcus aureus	99.5	HED5952428.1
ORF15	18854	19468	615	+	205	ATPase	Staphylococcus aureus	99.51	WP_150884669.1
ORF16	19465	19611	147	+	49	hypothetical protein	Staphylococcus aureus	100	WP_223223848.1
ORF17	19700	20149	450	-	150	hypothetical protein	Staphylococcus aureus	100	WP_001558060.1
ORF18	19700	20149	450	-	150	hypothetical protein	Staphylococcus aureus	100	WP_001558060.1
ORF19	20185	20370	186	-	62	hypothetical protein	Staphylococcus aureus	98.36	HDZ3343353.1
ORF20	20367	20513	147	-	49	hypothetical protein	Staphylococcus aureus	100	MBK4064412.1
ORF21	20525	21256	732	-	244	transcriptional regulator	Staphylococcus aureus subsp. aureus str. JKD6008	99.59	ADL66071.1
ORF22	21407	21646	240	+	80	helix-turn-helix transcriptional regulator	Staphylococcus aureus	100	MEW4671912.1
ORF23	21662	22438	777	+	259	Rha family transcriptional regulator	Staphylococcus aureus	99.61	WP_025176283.1
ORF24	22464	22907	444	+	148	hypothetical protein	Staphylococcus aureus	100	WP_031775158.1
ORF25	22904	23071	168	+	56	hypothetical protein	Staphylococcus aureus C2942	100	EZX41254.1
ORF26	23112	23324	213	-	71	uracil-DNA glycosylase	Staphylococcus aureus	98.57	HAR4446181.1

ORF27	23867	24010	144	-	48	hypothetical protein	Staphylococcus aureus	97.87	HDN3444135.1
ORF28	24000	24209	210	-	70	hypothetical protein	Staphylococcus aureus	100	MCE3415970.1
ORF29	24265	24510	246	+	82	transcriptional regulator	Staphylococcus phage phiSa2wa_st5	100	YP_010083638.1
ORF30	24479	24844	366	-	122	hypothetical protein	Staphylococcus aureus	99.17	WP_065316400.1
ORF31	24887	25114	228	+	76	hypothetical protein	Staphylococcus phage phiSa2wa_st1	98.67	YP_010083572.1
ORF32	25139	25402	264	+	88	DNA binding protein	Staphylococcus phage phiSa2wa_st1	100	YP_010083573.1
ORF33	25435	25545	111	-	37	DUF1270 domain-containing protein	Staphylococcus aureus	100	HCZ0996964.1
ORF34	25659	25982	324	+	108	transcriptional regulator	Staphylococcus aureus	99.07	HDE0419466.1
ORF35	25997	26359	363	+	121	hypothetical protein	Staphylococcus aureus	100	WP_031775157.1
ORF36	26356	27522	1167	+	389	DUF2800 domain-containing protein	Staphylococcus aureus	99.74	HDB3535692.1
ORF37	27548	28105	558	+	186	DUF2815 family protein	Staphylococcus aureus	100	HCY9553641.1
ORF38	28174	30126	1953	+	651	DNA polymerase	Staphylococcus aureus	100	WP_078100076.1
ORF39	30138	30293	156	+	52	transcriptional regulator	Staphylococcus aureus	100	HCZ5857143.1
ORF40	30293	30478	186	+	62	DUF3113 family protein	Staphylococcus aureus	98.36	EMB4689119.1
ORF41	30479	30838	360	+	120	SA1788 family PVL leukocidin-associated protein	Staphylococcus aureus	100	WP_000029380.1
ORF42	30839	31087	249	+	83	phi PVL orf 51-like protein	Staphylococcus aureus	100	HCZ0098113.1
ORF43	31128	31310	183	+	61	hypothetical protein	Staphylococcus aureus	100	WP_260595377.1
ORF44	31313	31714	402	+	134	hypothetical protein	Staphylococcus aureus	100	WP_000695760.1
ORF45	31907	32101	195	+	65	DUF1381 domain-containing protein	Staphylococcus aureus	100	MEO4287503.1
ORF46	32098	32301	204	+	68	hypothetical protein	Staphylococcus aureus	100	WP_031911912.1
ORF47	32294	32530	237	+	79	hypothetical protein	Staphylococcus aureus	100	HDD6704209.1
ORF48	32520	32909	390	+	130	hypothetical protein	Staphylococcus aureus	100	WP_001799187.1
ORF49	32906	33058	153	+	51	transcriptional activator	Staphylococcus phage Sa2wa-st88	100	UYL83430.1
ORF50	33093	33326	234	+	78	DUF1514 domain-containing protein	Staphylococcus phage vB_SauS_690	100	UKM36101.1
ORF51	33378	35825	2448	+	816	virulence-associated E family protein	Staphylococcus aureus	100	WP_000884847.1
ORF52	36106	36456	351	+	117	VRR-NUC domain-containing protein	Staphylococcus aureus	100	WP_353437336.1
ORF53	36437	37804	1368	+	456	DEAD/DEAH box helicase	Staphylococcus aureus	99.77	WP_078055707.1
ORF54	37817	38254	438	+	146	transcriptional regulator	Staphylococcus aureus	100	HDE6925101.1
ORF55	38411	38725	315	+	105	HNH endonuclease	Staphylococcus aureus	99.04	WP_113587697.1

ORF56	38836	39159	324	+	108	terminase small subunit	Staphylococcus phage phiSa2wa_st72	100	AUM57994.1
ORF57	39149	40840	1692	+	564	terminase large subunit	Staphylococcus aureus	99.82	WP_000153556.1
ORF58	40845	42083	1239	+	413	phage portal protein	Staphylococcus aureus	99.76	WP_031911915.1
ORF59	42067	42840	774	+	258	Clp protease ClpP	Staphylococcus aureus	99.22	HDE3394545.1
ORF60	42807	44015	1209	+	403	phage capsid protein	Staphylococcus aureus	98.76	QLI00759.1
ORF61	44084	44362	279	+	93	head-tail connector protein	Staphylococcus aureus	99.81	WP_106096736.1
ORF62	44374	44706	333	+	111	hypothetical protein	Staphylococcus aureus	99.09	HDI0152818.1
ORF63	44703	45104	402	+	134	hypothetical protein	Staphylococcus aureus	99.25	WP_147629992.1
ORF64	45105	45500	396	+	132	DUF3168 domain-containing protein	Staphylococcus aureus	99.24	WP_031775786.1
ORF65	45535	40360	642	+	214	phage tail protein	Staphylococcus aureus	99.53	HDA9240329.1
ORF66	46268	41826	456	+	152	Ig-like domain-containing protein	Staphylococcus aureus	100	WP_160200236.1
ORF67	46781	42215	351	+	117	phage tail assembly chaperone G	Staphylococcus aureus	100	WP_031791115.1
ORF68	47173	44621	159	+	53	hypothetical protein	Staphylococcus aureus	100	WP_353294710.1

79

Table S4. Comparison of the steady-state kinetic parameters for the oxidase-like activity of SapYZUs891@Fe/Mn-MOF and other nanozymes.

Catalyst	Substance	K_m (mM)	V_{max} [10^{-8} Ms^{-1}]	Reference
MOF-808	TMB	1.06	1.39	[10]
Fe-MOF@SalmpYZU47	TMB	1.2	25.3	[11]
Fe-N-C single-atom catalysts	TMB	1.81	0.601	[12]
graphitic carbon nitride	TMB	1.06	0.42	[13]
Fe_3O_4 -graphene oxide	TMB	1.12	0.13	[14]
Cu-MOF@PpZDSS02	TMB	4.03	24.49	[15]
EspYZU15@Pd	TMB	0.6	9.1	[16]
SapYZUs891@Fe/Mn-MOF	TMB	0.45	2.35	This study

Table S5. Comparison between SapYZUs891@Fe/Mn-MOF chromogenic system and previously reported studies.

Material	Method	Detection time (min)	Detection range (CFU/mL)	LOD (CFU/mL)	Ref.
SapYZUs891@Fe/Mn-MOF	Colorimetry	19	$3 \times 10^1 \sim 3 \times 10^8$	69	This study
IgG	ELISA	90	-	-	[17]
poly-L-lysine-functionalized magnetic beads (PLL-MB)	PCR	240	$1.8 \times 10^1 \sim 1.8 \times 10^6$	10^2	[18]
VAN-Au NPs/IgG	Lateral flow assay methods	-	$10^3 \sim 10^8$	10^3	[19]
Antibody/Nanopitted polyelectrolyte coatings	Long-period fiber grating	30	$10^4 \sim 10^8$	224	[20]
Vancocin/IgG	Fluorescence	130	1.0×10^3– 1.0×10^9	290	[21]
Antibody/AuNP/MNPs	Colorimetry	40	1.5×10^4–1.5×10^8	1.5×10^3	[22]
Aptamer	Localized surface plasmon resonance	2	-	10^3	[23]
Copper nanoclusters	Fluorescence	45	$10^2 \sim 10^8$	80	[24]
Nanobodies	Immuno-assay detection	480	$10^4 \sim 10^{10}$	1.4×10^5	[25]
AuPt@vB_YepM_ZN18	Colorimetry	40	$2.5\times 10^1 \sim 2.5 \times 10^5$	14	[26]
Cu-MOF@PpZDSS02	Colorimetry	46	$10^2 \sim 10^8$	3.3	[15]
AuNPs@T156	Colorimetry	80	3.8×10^1-3.8×10^9	38	[27]

Table S6. The effect of different food additives on the SapYZUs891@Fe/Mn-MOF chromogenic system in culture.

**Food additive**	**Spiked （CFU/mL）**	**Detected （CFU/mL）**	**RSD （%,n = 3）**	**Recovery (%)**	**PC （CFU/mL）**
braised beef with potatoes	1.35×10^{2}	1.85×10^{2}	1.83	121.48	1.65×10^{2}
	1.07×10^{4}	0.98×10^{4}	2.30	117.35	1.05×10^{4}
	0.97×10^{6}	1.05×10^{6}	0.76	109.09	1.31×10^{6}
curry chicken	1.26×10^{2}	1.24×10^{2}	1.87	93.85	0.71×10^{2}
	0.94×10^{4}	1.01×10^{4}	2.91	94.51	0.92×10^{4}
	0.63×10^{6}	0.72×10^{6}	0.57	98.06	1.02×10^{6}
stirfried pork	1.58×10^{2}	1.63×10^{2}	1.51	98.70	0.77×10^{2}
	1.02×10^{4}	1.06×10^{4}	0.81	109.23	0.71×10^{4}
	0.8×10^{6}	0.77×10^{6}	0.93	93.86	1.13×10^{6}
steamed duck with mushroom	0.85×10^{2}	0.88×10^{2}	2.69	98.94	0.92×10^{2}
	1.23×10^{4}	1.13×10^{4}	1.54	92.52	0.95×10^{4}
	1.55×10^{6}	1.40×10^{6}	0.85	110.66	1.19×10^{6}
black pepper beef	1.22×10^{2}	1.10×10^{2}	1.75	93.46	1.31×10^{2}
	0.85×10^{4}	0.9×10^{4}	0.87	93.50	1.09×10^{4}
	1.15×10^{6}	1.09×10^{6}	2.43	88.54	1.07×10^{6}

References

1. Han, Y.; Zhou, W.; Wu, Y.; Deng, A.; Yuan, L.; Gao, Y.; Li, H.; Wang, Z.; Wang, B.; Zhu, G.; et al. Characterisation of a colourimetric biosensor SapYZUM13@Mn_3O_4-NH_2 reveals the mechanisms underlying its rapid and sensitive detection of viable *Staphylococcus aureus* in food. Food Chem. **2024**, 457, 140189.
2. Zhang, L.; Zhang, W.; Nie, Y.; Wang, Y.; Zhang, P. Covalent organic framework-supported ultrasmall Rh nanoparticles as peroxidase mimics for colorimetric sensing of cysteine. J. Colloid. Interface. Sci. **2023**, 636, 568-576.
3. Isho, R.D.; Mohammad, N.M.S.; Omer, K.M. Enhancing enzymatic activity of Mn@Co_3O_4 nanosheets as mimetic nanozyme for colorimetric assay of ascorbic acid. Anal. Biochem. **2022**, 654, 114818.
4. Wang, D.; Gu, J.; Wang, H.; Liu, M.; Liu, Y.; Zhang, X. Promoting photoelectrochemical water oxidation of $BiVO_4$ photoanode via Co-MOF-derived heterostructural cocatalyst. Appl. Surf. Sci. **2023**, 619, 156710.
5. Lian, Q.; Zheng, X.; Peng, G.; Liu, Z.; Chen, L.; Wu, S. Oxidase mimicking of $CuMnO_2$ nanoflowers and the application in colorimetric detection of ascorbic acid. Colloid Surf. A-Physicochem. Eng. Asp. **2022**, 652, 129887.
6. Zhou, W.; Li, Y.; Zhu, G.; Xu, X.; Hu, Q.; Yang, Z.; Gu, X. High-sensitivity and high-specificity colorimetric detection of viable *Staphylococcus aureus* in ready-to-eat foods using a temperate-bacteriophage-based system with peroxidase-like activity. Sensors and Actuators B: Chemical. **2024**, 399, 134810.
7. Wang, X.; Liu, H.; Qiao, C.; Ma, Y.; Luo, H.; Hou, C.; Huo, D. A dual-functional single-atom Fe nanozyme-based sensitive colorimetric sensor for tannins quantification in brandy. Food

Chem. **2024**, 434, 137523.

8. Chen, M.; Zhou, H.; Liu, X.; Yuan, T.; Wang, W.; Zhao, C.; Zhao, Y.; Zhou, F.; Wang, X.; Xue, Z.; et al. Single iron site nanozyme for ultrasensitive glucose detection. Small. **2020**, 16, e2002343.
9. Wang, J.; Huang, R.; Qi, W.; Su, R.; He, Z. Construction of biomimetic nanozyme with high laccase- and catecholase-like activity for oxidation and detection of phenolic compounds. J. Hazard. Mater. **2022**, 429, 128404.
10. Zheng, H.Q.; Liu, C.Y.; Zeng, X.Y.; Chen, J.; Lu, J.; Lin, R.G.; Cao, R.; Lin, Z.J.; Su, J.W. MOF-808: a metal-organic framework with intrinsic peroxidase-like catalytic activity at neutral pH for colorimetric biosensing. Chem. **2018**, 57, 9096-9104.
11. Gao, L.; Zhang, L.; Yang, J.; Ma, T.; Wang, B.; Yang, H.; Lin, F.; Xu, X.; Yang, Z.Q. Immobilization of a broad host range phage on the peroxidase-like Fe-MOF for colorimetric determination of multiple *Salmonella enterica* strains in food. Mikrochim. Acta. **2024**, 191, 331.
12. Wu, Y.; Jiao, L.; Luo, X.; Xu, W.; Wei, X.; Wang, H.; Yan, H.; Gu, W.; Xu, B.Z.; Du D; et al. Oxidase-like Fe-N-C single-atom nanozymes for the detection of acetylcholinesterase activity. Small. **2019**, 15, e1903108.
13. Wu, Y.; Wen, J.; Xu, W.; Huang, J.; Jiao, L.; Tang, Y.; Chen, Y.; Yan, H.; Cao, S.; Zheng, L.; et al. Defect-Engineered Nanozyme-Linked receptors. Small. **2021**, 17, e2101907.
14. Song, Z.; Jiang, C.; Wang, F.; Yu, L.; Ye, S.; Dramou, P.; He, H. Nanozyme based on graphene oxide modified with Fe_3O_4, CuO, and cucurbit[6]uril for colorimetric determination of homocysteine. Mikrochim. Acta. **2021**, 188, 207.
15. Gao, L.; Long, Q.; Cen, B.; Gao, Q.; Tan, M.; Zhang, L.; Yang, J.; Ma, Y.; Xu, X.; Yang, Z.Q.

Immobilization of a novel bacteriophage PpZDSS02 onto the peroxidase-mimicking Cu-MOF for colorimetric sensing of *Proteus penneri* encompassing both promotion and inhibition mechanisms. Food Chem. **2025**, 472, 142887.

16. Zhang, Y.; Xu, X.; Yang, J.; Tan, M.; Zhou, W.; Gao, L.; Yang, Z. Directional immobilization of phage on the palladium-based nanozyme for colorimetric detection of *Cronobacter sakazakii* in powdered infant formula. Lwt. **2023**, 186, 115260.
17. Deng, A.; Li, X.; Fan, X.; Li, Y.; Han, Y.; Guan, T.; Wang, S.; Zhu, G.; Yang, Z.; Zhou, W. Rapid and interference-resistant colorimetric detection of viable *Staphylococcus aureus* in food using a broad host range phage SapYZU04 and MnFeO nanozyme. Food Control. **2025**, 171, 111101.
18. Deng, M.; Wang, Y.; Chen, G.; Liu, J.; Wang, Z.; Xu, H. Poly-l-lysine-functionalized magnetic beads combined with polymerase chain reaction for the detection of *Staphylococcus aureus* and *Escherichia coli* O157:H7 in milk. J. Dairy. Sci. **2021**, 104, 12342-12352.
19. Zhao, M.; Yao, X.; Liu, S.; Zhang, H.; Wang, L.; Yin, X.; Su, L.; Xu, B.; Wang, J.; Lan, Q.; et al. Antibiotic and mammal IgG based lateral flow assay for simple and sensitive detection of *Staphylococcus aureus*. Food Chem. **2021**, 339, 127955.
20. Yang, Z.Q.; Tao, X.Y.; Zhang, H.; Rao, S.Q.; Gao, L.; Pan, Z.M.; Jiao, X.A. Isolation and characterization of virulent phages infecting *Shewanella baltica* and *Shewanella putrefaciens*, and their application for biopreservation of chilled channel catfish (*Ictalurus punctatus*). Int. J. Food Microbiol. **2019**, 292, 107-117.
21. Kong, W.; Xiong, J.; Yue, H.; Fu, Z. Sandwich fluorimetric method for specific detection of *Staphylococcus aureus* based on antibiotic-affinity strategy. Anal. Chem. **2015**, 87, 9864-9868.
22. Sung, Y.J.; Suk, H.J.; Sung, H.Y.; Li, T.; Poo, H.; Kim, M.G. Novel antibody/gold

nanoparticle/magnetic nanoparticle nanocomposites for immunomagnetic separation and rapid colorimetric detection of *Staphylococcus aureus* in milk. Biosens. Bioelectron. **2013**, 43, 432-439.

23. Khateb, H.; Klos, G.; Meyer, R.L.; Sutherland, D.S. Development of a Label-Free LSPR-Apta sensor for *Staphylococcus aureus* detection. ACS Appl. Bio Mater. **2020**, 3, 3066-3077.
24. Pebdeni, A.B.; Mousavizadegan, M.; Hosseini, M. Sensitive detection of *S. Aureus* using aptamer- and vancomycin-copper nanoclusters as dual recognition strategy. Food Chem. **2021**, 361, 130137.
25. Hu, Y.; Sun, Y.; Gu, J.; Yang, F.; Wu, S.; Zhang, C.; Ji, X.; Lv, H.; Muyldermans, S.; Wang, S. Selection of specific nanobodies to develop an immuno-assay detecting *Staphylococcus aureus* in milk. Food Chem. **2021**, 353, 129481.
26. Yang, Q.L.; Wu, D.; Aziz, A.; Deng, S.S.; Zhou, L.; Chen, W.; Asif, M.; Wang, S.Q. Colorimetric platform based on synergistic effect between bacteriophage and AuPt nanozyme for determination of *Yersinia pseudotuberculosis*. Mikrochim. Acta. **2023**, 190, 76.
27. Wang, Y.; Wang, X.; Yan, Y.; Wang, J.; Lu, Y.; Abd El-Aty, A.M.; Wang, X. A visual colorimetric assay based on phage T156 and gold nanoparticles for the sensitive detection of *Salmonella* in lettuce. Anal. Chim. Acta. **2023**, 1272, 341501.
